# Tunable transcription factor library for robust quantification of regulatory properties in *Escherichia coli*

**DOI:** 10.15252/msb.202110843

**Published:** 2022-06-13

**Authors:** Vinuselvi Parisutham, Shivani Chhabra, Md Zulfikar Ali, Robert C Brewster

**Affiliations:** ^1^ Department of Systems Biology University of Massachusetts Chan Medical School Worcester MA USA; ^2^ Department of Pharmacological Sciences Icahn School of Medicine at Mount Sinai New York NY USA; ^3^ Department of Microbiology and Physiological Systems University of Massachusetts Chan Medical School Worcester MA USA

**Keywords:** bacterial physiology, genetic library, paralogs, quantitative gene regulation, transcription regulation, Chromatin, Transcription & Genomics, Methods & Resources

## Abstract

Predicting the quantitative regulatory function of transcription factors (TFs) based on factors such as binding sequence, binding location, and promoter type is not possible. The interconnected nature of gene networks and the difficulty in tuning individual TF concentrations make the isolated study of TF function challenging. Here, we present a library of *Escherichia coli* strains designed to allow for precise control of the concentration of individual TFs enabling the study of the role of TF concentration on physiology and regulation. We demonstrate the usefulness of this resource by measuring the regulatory function of the zinc‐responsive TF, ZntR, and the paralogous TF pair, GalR/GalS. For ZntR, we find that zinc alters ZntR regulatory function in a way that enables activation of the regulated gene to be robust with respect to ZntR concentration. For GalR and GalS, we are able to demonstrate that these paralogous TFs have fundamentally distinct regulatory roles beyond differences in binding affinity.

## Graphical abstract

This study presents a library of *Escherichia coli* strains where the concentration of individual transcription factors (TFs) can be independently tuned and measured, allowing for the analysis of copy number‐dependent TF roles in regulatory networks and cellular physiology.

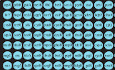

## Introduction

Transcription factors (TFs) are an important set of proteins that play a major role in controlling condition‐specific cellular decision‐making. Techniques such as DNaseI footprinting (Ellis *et al*, 2007), SELEX (Ishihama *et al*, 2016), ChIP‐seq (Galagan *et al*, 2013; Myers *et al*, 2015) and their variants have enabled high‐resolution base‐pair mapping of where TFs bind and which genes they control. However, predicting the direct regulatory effect of any given TF on a gene under its control remains challenging; the ability to build genetic circuits from natural TFs or foretell the regulation of promoters directly from its architecture is still completely lacking. One challenge to these predictions is the interconnected nature of regulatory networks. Individual TF genes typically regulate (and are regulated by) several to dozens of different genes and so controlling the concentration of a TF systematically and thus the quantitative regulatory function of that TF at a target is convolved with network and “context‐dependent” effects that hide the direct role of the TF on the gene. As a result, predicting the quantitative input‐output relationship between TF concentration and output of a gene based on regulatory architecture, i.e., the location, identity, and sequence of the TF‐binding sites that contribute to a promoters' regulation, is not possible. However, tremendous progress has been achieved towards the predictive design of gene circuits and network architectures using model TFs (Elowitz & Leibler, 2000; Gardner *et al*, 2000; Brewster *et al*, 2014; Nielsen *et al*, 2016; Potvin‐Trottier *et al*, 2016), although the toolbox of well‐characterized TFs is relatively sparse. Clearly, the characterization of a greater set of TFs would enable enhanced utility for biological engineering purposes while also deepening our understanding of why natural regulatory elements are built the way they are.

**Figure 1:**
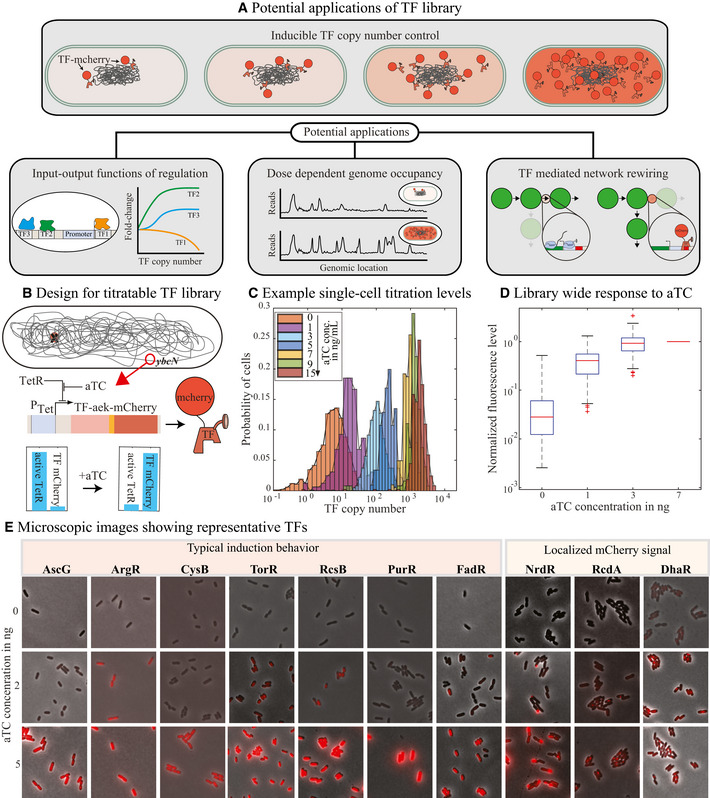
Design of titratable transcription factor library Potential applications of titratable TF library.Schematic representation of the titratable transcription factor library strain. TF is deleted from its native locus and expressed from the *ybcN* locus as a mCherry fusion construct and under the regulation of a tetracycline‐inducible promoter.Histogram represents single‐cell levels of TFs for increasing concentration of the inducer, aTC. Representative TF used here is RcsB.Box plot shows the distribution of mCherry levels for all the TFs in our library, at a given concentration of aTC. The central mark of the box plot is the median, the edges of the box are the 25^th^ and 75^th^ percentiles, the whiskers are the most extreme data points, and red symbols are the outliers. Two biological replicates were performed for each sample. Total number of cells analyzed per sample is listed in the source data file.Representative microscopic images showing different features of the strains as a result of TF titration. For instance, there is a significant change in length and mCherry signal for different levels of aTC in CysB library strains. DhaR, NrdR, and RcdA show localized mCherry signal. Source data are available online for this figure.

Here, we report the construction of a titratable copy of each of the 194 TFs in *Escherichia coli* for the purpose of characterizing the TF function. In this library, the copy number of any TF is controllable by induction rather than through indirect changes to growth or nutrient sources. The single‐cell TF level is also measurable due to a fusion of the TF with the mCherry fluorescent protein (Fig 1C). Importantly, the expression of the TF is isolated from the natural regulatory interactions that would limit or complicate copy number control. In addition, the titratable TF construct is stably integrated at a constant genetic locus in the chromosome to avoid any copy number difference. The ability to titrate TFs precisely enables a direct and quantitative measure of the role of a specific TF in regulation or physiology. Similar approaches with individual model TFs have enabled a deep understanding of the input‐output function of those specific TFs (Amit *et al*, 2011; Garcia & Phillips, 2011; Garcia *et al*, 2012; Jones *et al*, 2014; Sepúlveda *et al*, 2016; Chen *et al*, 2018; Einav *et al*, 2018). The resource introduced here enables studies of TFs as a whole with the same quantitative control typically dedicated to model TFs. When combined with systematically designed promoters, this library enables careful examination of the input‐output relationship of regulation for any TF in simple regulatory architectures that can reveal the fundamental regulatory function of these TFs. Overall, the goal of designing this resource is to enable detailed studies to characterize the regulatory function of TFs in a less biased way (Fig 1A).

In the remainder of the manuscript, we discuss the construction of the library followed by an account of the inducible TF copy number range of the library strains and the physiological impact of controlling the TF copy number in each strain. Finally, we give two examples of uses of the library to characterize the TF function. In the first vignette, we examine the unique regulatory architecture of heavy metal‐responsive TF, ZntR, and in the second, we examine the regulatory differences in a pair of paralogous TFs, GalR, and GalS. In both cases, we find that controlling the TF number enables unique insight into the fundamental regulatory behavior of these two groups of TFs.

## Results

### Transcription factor library

Construction of the titratable TF library is described, in detail, in the Materials and Methods section and in Fig 1B. Briefly, each of the 194 TF gene is deleted from its native locus, and the corresponding tf‐mCherry fusion gene (with an aek‐linker sequence (AEAAAKEAAAKA) separating the TF and mCherry genes), is expressed from a tetracycline‐inducible promoter and integrated at the *ybcN* locus. The inter‐protein linker would likely not interfere with the bio‐activity of the TF and enhance its stability (Arai *et al*, 2001). In addition, *tetR* gene is integrated at the *gspI* locus and the constitutive gene product (TetR) will repress the expression of the TF until the inducer, anhydrous tetracycline (aTC) is present. Upon addition of aTC, the TF gene is expressed and is tracked by measuring the mCherry levels at a given aTC concentration (histogram in Fig 1C shows the single‐cell level distribution of mCherry fluorescence for one representative TF, RcsB).

There are at least 198 genes of *E. coli* listed as transcription factors in RegulonDB (Santos‐Zavaleta *et al*, 2019). Our titratable TF library consists of 194 pairs (TF knockout and the corresponding titratable strain) of strains in total. The TFs included in our library can be classified into seven functional categories (Appendix Fig S2A): (i) transcriptional repressors (53 genes), (ii) transcriptional activators (39 genes), (iii) dual‐regulators (74 genes), (iv) histidine sensor kinase of the two‐component system (18 genes), (v) DNA‐binding regulator of the toxin/antitoxin system (eight genes), (vi) multi‐functional regulator (four genes), and (vii) pseudogenes (one gene). There are at least two pseudo TF genes (*gatR* and *glpR*) in *E. coli* genome. Of these two pseudogenes, *gatR* is inactivated by an “IS elementâ” inserted in the middle of the gene (Nobelmann & Lengeler, 1996), and as such it is not included in our library. On the other hand, for *glpR*, several genetic variants are reported in multiple rounds of sequencing. The *E. coli* MG1655 whole‐genome sequence listed in NCBI has a single nucleotide insertion causing a frame‐shift mutation. However, the *glpR* gene amplified from our lab stock of *E. coli* MG1655 does not contain this insertion (alleviating the frame shift), and hence that variant is included in our library. Despite repeated trials, the construction of *relE*, toxin gene, from a native toxin/antitoxin pair was unsuccessful. It is possible that even the “leaky” levels of RelE are enough to overwhelm the native expression of the toxin, RelB. As a result, *relE* and *relB* are also excluded from this library. Finally, the TF *alaS* is essential and the corresponding knockout is not available in the keio collection (Baba *et al*, 2006; Yamamoto *et al*, 2009) and, as such, we did not create a titratable strain for *alaS*.

Oftentimes, altering the growth condition is used as a way to control TF copy number in *E. coli* via changes in gene dosage, protein dilution, and network regulation (Kroner *et al*, 2019). However, these approaches do not always enable full control over a wide range of TF concentrations. For example, Appendix Fig S2D shows data from Schmidt *et al* (2016) measuring protein copy numbers over a wide range of growth conditions, it is clear that many TFs are not well controlled in this fashion, and oftentimes, growth rate is a poor predictor of TF copy number. By expressing TFs from a controlled tetracycline‐inducible promoter (at a common genetic locus), we eliminate many of the native transcriptional regulatory network features that otherwise influence the number of TFs and create a precise control of the TF copy number through induction (Fig 1D and Appendix Fig S2E).

### Quantification of TF copy number

**Figure 2:**
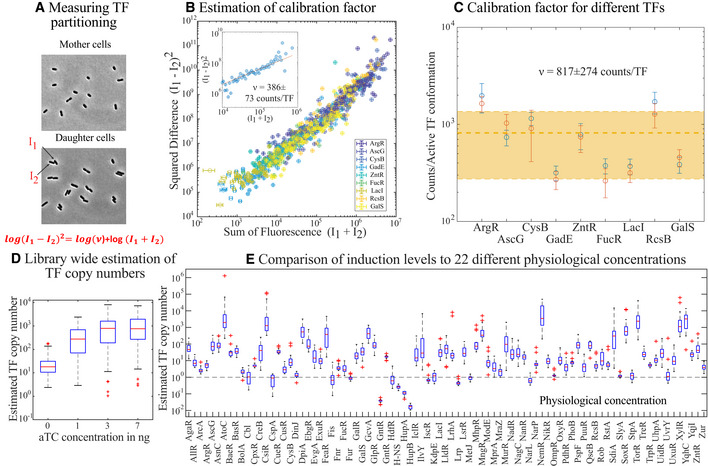
Estimations of TF number Representative images taken before (top) and after (bottom) one cell division in order to calculate the stochastic fluctuation in fluorescence distribution between the daughter cells, *I*_1_ and *I*_2_.Plot showing the sum (*I*_1_ + *I*_2_) and squared difference ((*I*_1_−*I*_2_)^2^) in fluorescence of the daughter cell pairs for nine different TFs. Refer to Appendix Fig S5 for raw data and binned values for individual TFs. Two different replicate measurements are performed per TF strain.Measured calibration factor for nine different TF strains in our library. Blue and red points correspond to two different replicates. Error bar represents 95% confidence interval of individual fits. The dashed line and shaded area represent the mean value and standard deviation of the calibration factor for all TFs. Source data are available online for this figure.The data in Fig 1D are converted to absolute TF numbers using the estimated mean calibration factor.Comparison of the TF copy number at maximum induction with the measured protein copies/cell in the work by Schmidt *et al* (2016). For the box plots in (D and E), the central mark is the median, the edges are the 25^th^ and 75^th^ percentiles, the whiskers are the most extreme data points, and the red symbols are the outliers. Two biological replicates were performed for each sample. Source data are same as that used in Fig 1D. Source data are available online for this figure.

The mCherry fusion allows for the direct quantification of absolute TF copy number based on the proportionality between fluorescent signal (*I*) and the number of fluorophores (*N*), *I* = *vN*. The arbitrary fluorescence signal measured from a microscope or flow cytometer can be converted to the number of fluorescent proteins by estimating the calibration factor, *v*. The techniques to measure *v* typically involve measuring either the fluorescence of a single molecule in photobleaching experiments (Weiss, 1999; Sugiyama *et al*, 2005; Garcia *et al*, 2011) or the measurement of a larger molecule that contains a fixed number of fluorescent protein (Dundr *et al*, 2002; Cherkas *et al*, 2018). An alternative method for quantifying TF copy number from fluorescence signal involves measuring fluctuations around the mean of a stochastic event that involves the fluorescent protein; two examples include measuring fluorescence level differences of two daughter cells immediately after division (Fig 2A; Rosenfeld *et al*, 2006; Brewster *et al*, 2014) or measuring fluctuations in the fluorescent bleaching trajectory of fluorophores in a single‐cell (Nayak & Rutenberg, 2011; Kim *et al*, 2016; Bakker & Swain, 2019). In our library strains, we used fluctuations in the partitioning of fluorescence signal between the daughter cells (*I*_1_ and *I*_2_) to measure the calibration factor for nine strains (see Fig 2B and Materials and Methods “Estimation of calibration factor”) and hence the number of TFs. The estimate of *v* is based on the assumption that the TF‐mCherry proteins are randomly distributed between the two daughter cells upon division. In general, we find that the calibration factor for each TF is similar (within ± 2‐fold of the mean of the calibration factor for the nine TFs, see the yellow shaded region in Fig 2C). Determining the calibration factor for all 194 TFs would be laborious and the actual value of *v* depends heavily on experimental settings (microscope optics, exposure times, etc), we chose to use the mean of the nine measured calibration factors to estimate the number of TFs in each strain throughout the library. However, it is important to note that there may be cases where this estimate is significantly off. As such, the measurement of the TF number for other strains should be thought of as an estimate; if precise knowledge of TF numbers in a specific strain is required, an individual measurement in that specific library strain should be made. In Fig 2D, we show the response of the titratable library strains to aTC. We observed a 100–1,000 fold increase in TF numbers (or simply mCherry signal intensity) as aTC concentration is increased (Fig 2D). In Fig 2E, we compare the maximum induction level of TFs in our library to the measured TF numbers per cell, under 20 different growth conditions for *E. coli* from the work of Schmidt *et al* (2016). The vast majority of these data points are above one, indicating that our induction strains are capable of reaching to and beyond the physiological concentrations of most TFs.

### Physiological effects of TF titration

**Figure 3:**
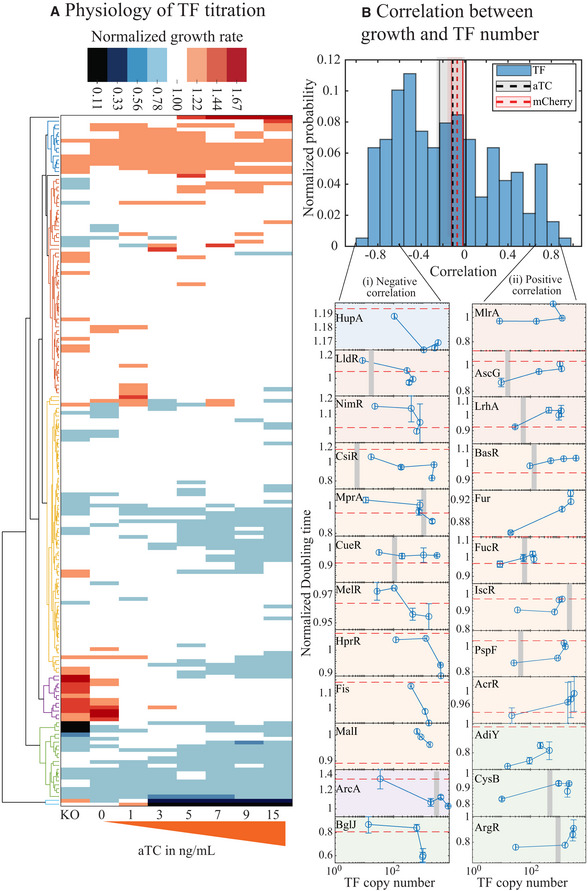
Physiology of the titratable library strain Cluster analysis of growth rates of the library strain in different concentration of aTC. The growth rate used here is normalized to the growth rate of wild‐type MG1655 measured in similar aTC concentration. (Refer to Appendix Fig S8 for individual clusters and their corresponding labels).Histogram showing the correlation between growth rates and the estimated TF copy number. Shown in gray shades is the correlation for wild‐type MG1655 in different aTC concentration, and shown in red shades is the correlation between growth rates and total mCherry. Lateral panel shows representative examples of strains showing high correlation between growth rates and TF copy number. The gray bar represents physiological TF copies/cell measured by Schmidt *et al* (2016). The panels are colored to match the grouping in the clustergram in (A). The red dashed line represent growth rate of corresponding TF knockout strain. Error bar for growth rate is the standard deviation of three biological replicates, and the error bar for TF copy number is the standard error calculated from single‐cell data. Total number cells analyzed are listed in the source table. Source data are available online for this figure.

Transcription factors are directly (or indirectly) involved in rewiring the function of clusters of genes within the cell. Clearly, there will be physiological consequences for altering the concentration of some of the TFs. For instance, some TFs such as Crp, ArgR, CysB, and MetJ are critical for essential metabolic pathways, and under‐producing or deleting these TFs may seriously affect the fitness of the corresponding strains under certain induction conditions. On the other hand, some TFs such as Nac and PdhR may be toxic when expressed at high concentrations (Mediati *et al*, 2018). The physiological effects of different TFs might be influenced by the growth media (for instance, in Appendix Fig S7 shows the impact of different carbon sources on the growth of the library strain expressing H‐NS). Here, we will use the growth rate in glucose minimal media as a proxy to evaluate the fitness or physiological effects due to the titration of each TF. The steady‐state growth rate of each TF library strain is measured in different aTC concentrations and normalized to the growth rate of wild‐type in the corresponding aTC concentration. Hierarchical cluster analysis results in six major clusters of growth phenotypes (Fig 3A). Furthermore, we calculate the correlation coefficient between growth rate and aTC concentration for each library strain (Fig 3B). The correlation coefficients are roughly trimodal with one peak around negative correlation values, one near zero, and the final less‐defined peak for positive correlations. The majority of strains show a negative growth correlation with aTC. Importantly, this is not due to aTC toxicity; the gray‐shaded box in Fig 3B shows the correlation between wild‐type growth rate and aTC concentration. We also validate that there is no correlation between total mCherry levels and the growth rate (red shaded box, Fig 3B). It is evident that the higher correlation between TF copy number and growth rates is primarily due to the physiological consequence of TF expression and not due to protein overexpression or aTC toxicity.

In Fig 3A, the top‐most cluster in blue corresponds to TF strains that grow faster than wild type at most aTC concentration. A few genes of this cluster including McbR, BluR, and CsgD are involved in regulating biofilm formation. PurR, a regulator of purine metabolism, exhibits the fastest growth rate with a doubling time of 41 ± 2 min. The second cluster, in red, has two bigger nodes. Top nodes include TFs whose knockouts grow slower than or equal to wild‐type and TF titration helps enhance the growth rate. Some of the TFs in this cluster exhibit a positive correlation between TF copy number and growth rate (Fig 3B, lateral panel (ii): MlrA, AscG, and LrhA). The bottom node includes TFs with knockouts growing faster than wild type and TF titration retards the growth rate exhibiting a negative correlation between TF copy number and growth rate (Fig 3B, lateral panel (i): LldR, and NimR). The third cluster includes TF strains that show overall reduced growth compared with wild type. The slowest doubling time in this cluster is 89 min for the TF, MntR involved in sensing heavy metal, manganese. In this cluster, the growth rates across different aTC concentration are fairly constant; however, there are exceptions (such as Fig 3B, lateral panel (i): MalI, Fis, HprR, and lateral panel (ii): Fur, PspF, and AcrR) where we see a good correlation between growth rate and TF copy number. The fourth cluster in purple includes TFs with knockouts and the lowest aTC concentrations growing faster than the wild type. Further increase in aTC causes a reduction in growth rate (Fig 3B, lateral panel (i): ArcA). Strains with extreme growth defects are part of the green and cyan clusters. In the first node of the green cluster are TF strains where the knockout shows "no growth" or reduced growth and expression of just enough TF is sufficient to rescue the growth rate. All TFs of the amino acid metabolic pathway (ArgR, CysB, MetR, MetJ, and LysR) belong to this cluster. In the second node are TF strains, which show a drastic decrease in growth rate upon TF titration. The slowest doubling time measured in this cluster is 115 min for the TF, BglJ (Fig 3B, lateral panel (i): BglJ). Cyan cluster has only two strains, Nac and PdhR, that stop growing beyond the 3 ng/ml of aTC in the medium. In summary, Nac and PdhR are the only two TF strains exhibiting severe growth defects hampering their use in the titratable TF expression.

### Case studies using TF titration library

In the following two sections, we demonstrate how the TF titration library can be used to dissect the regulatory function of individual TFs. The library is particularly effective when combined with other genetic resources that allow for systematic control of promoter architectures. The vignettes of these sections make use of two reporter libraries, the Zaslaver's transcriptional reporter library (Zaslaver *et al*, 2004) and a “TF‐binding position library” from our lab which enables controlled movement of a TF‐binding site on a synthetic promoter (preprint: Guharajan *et al*, 2021).

### Case study 1: Regulation by the zinc‐responsive TF, ZntR

**Figure 4:**
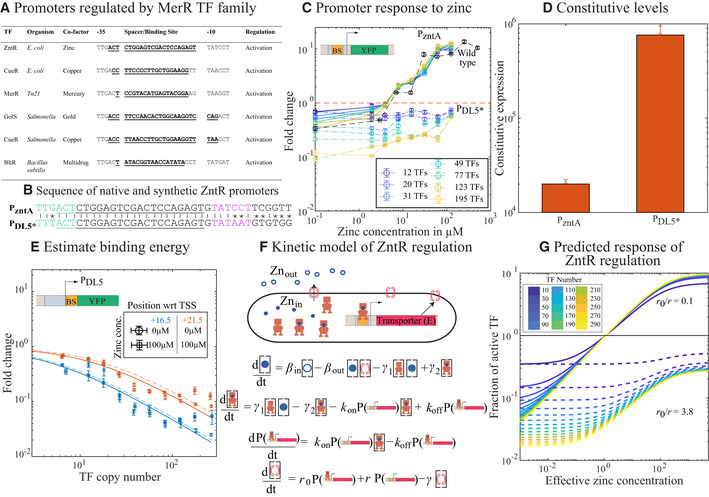
Regulation mediated by ZntR and zinc Table showing the signature regulatory motif of the common MerR TF families in different bacteria. TFs of the MerR family have their binding site acting as a spacer between the −35 and −10 boxes for RNAP binding.Shows the sequence of *P*_zntA_ and P_DL5*_ with ZntR‐binding site as the spacer. Shown in green is the −35 element and in magenta is the −10 element. The sequence of ZntR binding site is underlined.Response curve for the *P*_zntA_ promoter in wild‐type (black dashed line) and in ZntR library strains (with fixed TF concentration (solid line)) for different concentration of zinc. The dotted lines are the response of the modified P_DL5*_ promoter with ZntR‐binding site as the spacer. TF numbers in the legend represent the mean of the TF concentration from the binned value for the corresponding curve.Constitutive values of *P*_zntA_ and P_DL5*_ measured in ZntR knockout strain.Regulatory curves when the binding site for ZntR is downstream of the native promoter of *P_DL5_*. The ZntR‐binding site acts as pure repressor independent of the zinc concentration when present outside the core promoter element.Simple kinetic model used to decipher the key regulatory features of ZntR–zinc‐mediated regulations. The reporter here is the transporter gene, E. *r*_0_ is the basal expression from the promoter for E, and *r* is the acceleration brought about when the promoter for E is bound by an active TF (TF* or zinc–TF complex).The output of the kinetic model when the ratio of *r*_0_/*r* is altered. When *r*_0_ is less than *r*, there is repression at lowest zinc concentrations that is dependent on TF copy number and activation at higher concentrations similar to the response form *P*_zntA_ promoter (solid lines in C). When *r*_0_ is greater than (dotted line) *r*, there is strong repression and weak activation, and this regulatory feature is similar to that observed for P_DL5*_ promoter with ZntR‐binding site (dotted line in C). Note that we only make qualitative match between the model and the experimental data. For all the experimental results associated with this figure, single‐cell fold change is calculated independently for the three biological replicates, and all the single‐cell data are binned. Each data point corresponds to the mean of single‐cell data in the given bin, and error bars are the standard error of the data points in the given bin. Source data are available online for this figure.

The architecture of a promoter (i.e., the position, identity, and specificity of TF‐ and RNAP‐binding sites) is a fundamental indicator of the overall regulatory activity of a gene. Elucidating the mechanisms of common promoter architectures can help lay ground rules to build well‐defined genetic parts. For instance, the architecture of promoters involved in sensing heavy metals such as copper, zinc, gold, and mercury are very similar across different bacterial species (Fig 4A). Interpreting the biophysical constraints of such promoter architectures will help in different biological applications such as in whole‐cell biosensors. The common promoter architecture of heavy metal‐responsive genes involves a single TF‐binding site acting as the spacer between the −10 and −35 promoter sequence making the length of the spacer sequence unusually longer, i.e., 19–20 bp, whereas the optimal spacer length for *E. coli σ* ^70^ promoter is 17 bp (Ansari *et al*, 1992; Yona *et al*, 2018; Fig 4A). This architecture is common for the MerR family of TFs in *E. coli* (such as the metal‐responsive TFs, *cueR,* and *zntR*, responding to copper and zinc, respectively) and is also found in TFs from other organisms such as *bltR* of *Bacillus* and *merR* of transposable elements (Brown *et al*, 2003; Fig 4A). According to previous studies, the mechanism of activation of these promoters involves a distortion of the promoter DNA upon binding of the co‐factor bound TF which can realign the −35 and −10 boxes and recruit RNA polymerase in order to initiate transcription. However, these studies involved mutating or truncating the spacer sequence which in turn will abolish the TF‐binding site (Brown *et al*, 2003). Under this context, the precise input‐output function of these types of promoters can often be difficult to tease apart, hence we examined the zinc‐responsive TF, ZntR from our library, as a model TF to understand the properties of MerR family of TFs. The only known function of ZntR is in regulating the expression of ZntA, a transmembrane protein that mediates the export of zinc and other heavy metals. Less is known about the regulations of ZntR in *E. coli*, although according to the proteomic studies by Schmidt *et al* (2016) the concentration of ZntR is roughly 40–60 copies/cell under different physiological conditions. ZntR uses zinc as a co‐factor, although it also can weakly recognize other heavy metals like cadmium (Brocklehurst *et al*, 1999). Accumulation of zinc (and other heavy metals) inside the cell could be deleterious to the cell hence, we want to keep the native ZntA (zinc exporter and the only known target gene for ZntR) intact and just clone the *P*_zntA_ promoter to a reporter plasmid (low copy SCS101 plasmid maintained at roughly four copies per cell; Shao *et al*, 2021) to study the regulatory profile of ZntR. Using the titratable TF library strain for ZntR along with the *P*_zntA_ reporter plasmid, we are able to independently control both the co‐factor and the TF copy number while quantitatively measuring *P*_zntA_ regulation.

In line with the ground rule for the common genetic architecture of the MerR family of TFs (Fig 4A), the *P*_zntA_ promoter contains the binding site for ZntR as the spacer sequence between the −35 and −10 box for RNAP binding (Fig 4B). The length of spacer sequence in this promoter is 19 bp. Measurements for constitutive expression (in ZntR knockout strains) from the *P*_zntA_ promoter are relatively very low. To understand the natural response function of ZntR to activate the *P*_zntA_ promoter, we measured the response of promoter *P*_zntA_ to different zinc concentrations in a standard laboratory strain (MG1655), as seen from the black dashed curve in Fig 4C. We see that ZntR has a zinc‐dependent regulatory function that is not simply “inactive” to “active” but instead it changes from a repressor to an activator with zinc. It is important to note that we only control zinc concentration in the media rather than the intracellular concentration, in practice the levels of internal zinc are complex, as the primary zinc exporters are changing with zinc and zinc is being bound by several metallo‐regulatory proteins in the cell including RNAP (Chanfreau, 2013). However, the *P*_zntA_ promoter clearly responds to the titration of external zinc and shows repression at low zinc levels and activation at higher zinc concentrations with a maximum fold change of 10 when compared to the expression from a *zntR* knockout strain at the same zinc concentration. We next tested the effect of zinc titration in our library strain for several fixed TF copy numbers (solid color curves in Fig 4C). Repression of the *P*_zntA_ promoter by ZntR can be small (roughly 10% or so) for low concentrations of TF and up to roughly 2‐fold in the presence of hundreds of TFs. Our library strains reveal a very interesting feature of this circuit; while the repression levels seen at low zinc concentrations strongly depend on the number of TFs in the cell, activation of the promoter by the TF is largely independent of the total number of TFs; the curves collapse for concentrations above roughly 1 µM of zinc (also see Appendix Fig S3A for a plot showing fold change as a function of TF copy number for varying zinc). This means that the overall activated response of this system is robust to the TF level.

As described above, regulation by the MerR family of TFs is thought to be accounted for the unusual spacer length. We wanted to examine ZntR regulatory function on a stronger promoter with an ideal spacer length. In Fig 4B, we show how we integrated the ZntR‐binding site as a spacer sequence into a common synthetic promoter derived from the *lac* operon, *P_DL5_* (Brewster *et al*, 2012); replacing the spacer sequence of *P_DL5_* with those from *P*_zntA_ creates a promoter, P_DL5*_. Constitutive values (in the absence of ZntR) from P_DL5*_ are 40 times higher than from *P*_zntA_ (see Fig 4D). Importantly, the −35 and −10 boxes of P_DL5*_ are very similar to the *P*_zntA_ promoter with only a single nucleotide change in the −35 box and two changes in the −10 box (see “asterisk” signs between the sequences in Fig 4B). The data for the regulation of P_DL5*_ by ZntR are shown as dotted lines in Fig 4C; we once again see repression for a given number of TFs, which is alleviated by zinc. The addition of zinc relieves the repression of P_DL5*_ slightly, but we no longer see clear activating behavior, i.e., fold change did not increase above 1.

Next, we questioned the regulations of ZntR at locations other than being the spacer sequence for the promoter. We measured ZntR regulatory function at two locations immediately downstream of the promoter (centered at +16.5 and +21.5). From previous work on a handful of TFs, we anticipate that TFs regulate at these locations through pure steric hindrance where the fold change will be repressive and a reflection only of a single parameter, the occupancy of the TF (Ptashne *et al*, 1980; Ackers *et al*, 1982; Brewster *et al*, 2014; Forcier *et al*, 2018; preprint: Guharajan *et al*, 2021). In Fig 4E, we show the fold change for these two binding locations as a function of TF copy number both without added zinc (solid line) and with 100 µM zinc (dashed line). Significantly, the regulation both with and without zinc is the same, which implies that the presence of zinc does not alter the affinity of the TF for the binding site; this also suggests that the regulatory shift of ZntR with zinc at the native binding location emanates from a change in the regulatory function of the bound TF rather than a change in its occupancy. Fitting a simple steric hindrance model of regulation (described in the next subsection for “Regulatory role of paralogous TFs”) to these data enable measurement of the TF‐binding affinity (Garcia & Phillips, 2011), which is the sole‐free parameter of the thermodynamic model. We find a binding affinity of −13.8*k*_BT_, roughly equivalent in strength to LacI binding to the LacO2 operator (Brewster *et al*, 2014). Clearly, the regulatory function of ZntR, even in this small number of examples (as spacer sequence for a stronger or weaker promoter or at the steric hindrance location on a stronger promoter), is incredibly flexible; naturally, it is capable of providing zinc‐dependent regulation that switches function from repression to activation depending on zinc, but it also can serve at unique locations with respect to the promoter, as a TF whose function is insensitive to zinc availability.

To explore which attributes of the regulatory system enabled key features such as the robust TF number‐independent activation or TF‐dependent repression seen in Fig 4C, we built a simple kinetic model. The basic features of the model are detailed in Fig 4F and in the Materials and Methods section. The basal expression of the target gene occurs at rate *r*_0_ in the absence of the TF and when the TF‐zinc complex is bound, it acts as an activator increasing expression to a rate *r*. However, when zinc‐free TF is bound, it acts as a repressor capable of shutting off expression entirely. Zinc import is proportional to the external concentration whereas export is proportional to the concentration of ZntA in the cell. The results of Fig 4E allow us to estimate the binding energy and hence the ratio of on and off rates of DNA binding of ZntR ($k_{\text{on}}/k_{\text{off}}=\exp\left(‐\Delta \varepsilon \right)/N_{\text{ns}}$), which we set equal regardless of TF‐state (bound or unbound by zinc; Refer to Appendix Fig S3B for model behavior when $k_{\text{on}}/k_{\text{off}}=\exp\left(‐\Delta \varepsilon \right)/N_{\text{ns}}$ is altered). We solve this model for steady‐state and calculate the fold change of the target gene (the zinc transporter itself) as a function of effective zinc concentration (see Materials and Methods for the description of effective zinc concentration). The kinetic parameters used in the model are listed in Appendix Table S1. The steady‐state solutions require only two free parameters to account for (*r*_0_ and *r*) while other parameters could be derived from the experiments (*k*_on_/*k*_off_ and *γ*). Interestingly, when we consider the role of different promoters in this problem, we associate changes to the promoter with changes to *r*_0_ and possibly *r*. Since, we did not alter both the sequence and the location of the TF‐binding site (proxy for not altering the rate of activation by TF, *r*), we kept the value of *r* fixed and changed the basal rate *r*_0_ proportionately. We plot to fold change as a function of effective zinc concentration for different ratios of *r*_0_ and *r* in Fig 4G. The solid lines in Fig 4G show many of the important features from our measurement of *P*_zntA_ regulation; we see TF‐dependent repression and activation that is mostly copy number‐independent. Changing the promoter to P_DL5*_, we expect *r*_0_ to increase 38 fold (see Fig 4D). As shown in the figure the model predicts that the TF can switch between the two observed behaviors (strong to weak repression or strong repression to strong activation) simply by tuning the rate of basal expression (*r*_0_) relative to the active TF‐bound rate of expression *r*. This implies that ZntR acting on a strong promoter will exhibit stronger repression/weaker activation whereas a weaker promoter will exhibit weaker repression/stronger activation. Consistent with our model the measured constitutive value for P_DL5*_ is roughly 35–40 fold higher than for *P*_zntA_ Fig 4D. The dash lines in Fig 4G show the predictions from our model for regulation of P_DL5*_ assuming the only change to the system is in *r*_0_. while this does not perfectly capture the P_DL5*_ data from Fig 4C (specifically at low zinc) qualitatively the two responses are similar. We introduced random mutations to the P_DL5*_ in the −35 and −10 boxes and consistent with our model, we see weaker repression/stronger activation for mutations that weaken constitutive expression and stronger repression for mutations that strengthen the constitutive expression (see Appendix Fig S3C). These data clearly capture an interesting feature of the TF; the promoter‐specific behavior did not require a fundamental change in TF function, only in the basal expression of the promoter.

### Case study 2: Regulatory role of paralogous TFs

The phenomenon of paralogous TFs, TFs within an organism that arises from gene duplication and divergence, is common throughout living systems (Reece‐Hoyes *et al*, 2013; Voordeckers *et al*, 2015). Bacteria are particularly susceptible to this thanks to their propensity for lateral and horizontal gene transfer. Isorepressors are groups of TFs that share higher homology in the DNA‐binding domain and form overlapping regulons such that they regulate identical (or similar) sequence motifs. Some examples from *E. coli* include pairs of TFs such as GadW and GadX, and GalR and GalS but examples of isorepressor trios, also exist such as MarA, SoxS, and Rob. Although these TF sets recognize similar consensus sequence motifs, their actual regulatory functions may differ at both quantitative and qualitative levels.

**Figure 5:**
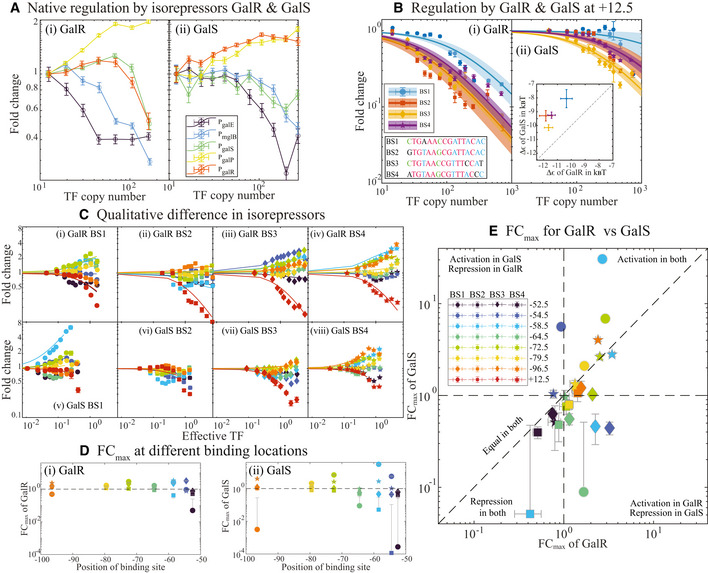
Quantitative and qualitative differences between the isorepressors GalR and GalS Plot showing the response of GalR (i) and GalS (ii) to native promoters regulated by the isorepressors. Refer to Appendix Fig S4A for direct comparison of regulation by GalR and GalS.Regulatory curves for binding sites (BS1, BS2, BS3, and BS4) in GalR (i) and GalS (ii) at position +12.5 relative to the TSS. The insert in (i) shows the binding sequence of the four motifs analyzed in this study. Nucleotide common to all four binding sites are in red. Nucleotide found in at least three sequences are in blue and in at least two sequences are in green. Unique nucleotide is in black. The insert in (ii) shows the inferred‐binding affinity for different binding sites in GalR and GalS.Plot showing fold change as a function of effective TF concentration, $N_{\text{TF}}\exp\left(‐\Delta \varepsilon \right)/N_{\text{ns}}$. Analysis of the regulatory curves for BS1, BS2, BS3, and BS4 at different locations (−52.5, −54.5, −58.5, −64.5, −72.5, −79.5, −96.5, and +12.5) relative to the TSS indicate different qualitative features of the TFs at different binding location.FC_max_ values at different binding locations for GalR (i) and GalS (ii).Shows a comparison of FC_max_ values of GalR and GalS at a given binding location. Data in each quadrant represent the unique features of GalR and GalS for the four binding sites. The lower quadrant (showing repression in both) is extended to Appendix Fig S4D, to show locations with very low FC_max_. The FC_max_ corresponding to +12.5 is zero. For all the experimental results associated with this figure, single‐cell fold change is calculated independently for the three biological replicates, and all the single‐cell data are binned. Each data point corresponds to the mean of single‐cell data in the given bin, and error bars are the standard error of the data points in the given bin. Error bars on the fits correspond to the 95% confidence interval of the fit. Source data are available online for this figure.

Since our library strains can be extended to allow multiple single‐gene deletions, we aimed to measure the differences in regulation between isorepressors, GalR, and GalS. To do this, we knocked out the paralogous TF in each of the GalR and GalS library strains (i.e., we knocked out GalR in the GalS library strain and vice versa). We then measured five native GalR‐GalS responsive promoters in these strains at a range of induction levels. The native promoters each have between 1 and 6 binding sites for GalR/GalS. The response of each of these promoters to titration of GalR or GalS is shown in Fig 5A. We find that four of these promoters have qualitatively similar responses to GalR and GalS, although the difference in magnitude of each response varies slightly with some promoters. On the other hand, the promoter for GalR (orange curve, Fig 5A), is activated with GalR and repressed with GalS. Interestingly despite both TFs being categorized as repressors, we see significant levels of activation in response to GalR and GalS for some of these promoters especially, *P*_galP_ (yellow curves, Fig 5A). It is perhaps unclear from the data in Fig 5A if the difference in regulation arises from the distinct TF function of each Gal paralog or if it is simply the result of differential affinity for the promoters.

We next tested four distinct binding site sequences; two sites that natively regulate *P*_galP_ (BS1 and BS2), one site from *P*_galS_ (BS3), and the last from *P*_galR_ (BS4) for regulatory differences in response to GalR or GalS. The natural location of these sites is at −243.5 for BS1, −61.5 for BS2 and BS3, and +8.5 for BS4. We measured fold change for each individual GalR/GalS‐binding site placed at different locations along with a synthetic promoter. In this case, the binding site we introduce represents the only known TF‐binding site on the promoter. Similar to our approach with ZntR, we first introduce each binding site directly downstream of the promoter. These data are shown in Fig 5B. The data for both TFs at each of the four binding sites fit well to the pure steric hindrance model of regulation allowing us to infer the binding energy of each sequence for either TF. The inset to Fig 5B compares these affinities and demonstrates that for each site GalR binds stronger than GalS. Interestingly, though, the rank order of sites is different for the two TFs; for both TFs BS1 is the weakest followed by BS4; however, BS2 is the highest affinity site for GalR while BS3 is the highest affinity site for GalS. These results indicate that the binding consensus for GalR and GalS are not the same.

Next, we infer the regulatory effect of GalR/GalS at other binding locations on the promoter. For this, we use a general model of gene regulation that quantifies the regulatory role of the TF (preprint: Guharajan *et al*, 2021),

(1) $$\text{Fold}\;\text{change}\;=\;\frac{1+{\text{FC}}_{\max}\exp\left(‐\Delta \varepsilon \right)N_{\text{TF}}/N_{\text{ns}}}{1+N_{\text{TF}}\exp\left(‐\Delta \varepsilon \right)/N_{\text{ns}}}\;,$$

where FC_max_ represents the maximum fold change at saturating TF concentration, exp(‐Δε) is the binding affinity measured as in the previous plots and $N_{\text{TF}}\exp\left(‐\Delta \varepsilon \right)/N_{\text{ns}}$ is the effective TF concentration, i.e., TF copy number normalized by the binding site affinity. Importantly, the steric model fit to that data are recovered by setting FC_max_ = 0. Figure 5C shows the fold change as a function of TF copy number for both GalR and GalS binding to each of the four binding sequences at binding locations upstream ranging from −52.5 to −89.5. Each of these data sets fits well to the model with FC_max_, the regulatory function of the TF, as the sole fit parameter. Figure 5D(i,ii) show the regulatory role of each binding site for GalR (i) and GalS (ii) as a function of binding location. Surprisingly, we see several cases where one binding site at a position causes activation whereas a different sequence causes repression (i.e there are points both above and below the black dashed line at 1 in Fig 5D(i,ii)). Figure 5E compares the regulatory effect for GalR and GalS. Data points fall in one of the four quadrants of this plot, the upper right and bottom left quadrants are for binding sequences that have the same qualitative role for GalR and GalS regulation at that location. On the other hand, points in the top left and bottom right quadrants have different qualitative regulatory functions for GalR and GalS; we see that the activation by GalR and repression by GalS is the most common form of differential regulation between the two TFs. Finally, data points that fall along the one‐to‐one dashed line have an identical quantitative regulatory function with either GalR or GalS. Clearly, despite having similar binding recognition, GalR and GalS can function as qualitatively different proteins beyond merely a preference for a given binding sequence and this difference depends on both binding sequence and binding location.

## Discussion

Genetic libraries have become an essential experimental resource in functional genomics. The primary goal of such genome‐wide mutant collections is the unbiased study of all genes to reveal how each is involved in the dynamics and robustness of a given cellular process. In particular, there is a rich assortment of such libraries available in *E. coli* including a single‐gene deletion library (the keio collection Baba *et al*, 2006; Yamamoto *et al*, 2009), a transcriptional reporter library (the Zaslaver library; Zaslaver *et al*, 2006), and the ASKA open reading frame clones (the ASKA library; Kitagawa *et al*, 2005), among others. These mutant libraries have served as a stand‐alone tool to address fascinating biological problems. For example, the keio deletion library has helped identify essential and nonessential genes across different growth conditions and facilitated the reconstruction of metabolic networks with higher precision (Orth *et al*, 2011; Fuhrer *et al*, 2017) and the transcriptional reporter library has been instrumental in obtaining the precision and dynamics in promoter activity under different extracellular perturbations (Zaslaver *et al*, 2004). Similar libraries have been constructed in other organisms; for instance, single‐gene deletion libraries exist for many bacteria such as *Bacillus subtilis* (Koo *et al*, 2017), *Pseudomonas aeruginosa* (Jacobs *et al*, 2003), and *Acinetobacter baylyi* (Berardinis *et al*, 2008) and also eukaryotic organisms such as *Saccharomyces cerevisiae* (Smith *et al*, 2011; Chong *et al*, 2012; Giaever & Nislow, 2014). Importantly, these libraries have also played crucial roles in unexpected ways, for instance the keio library has been an essential tool in the study of modulating host‐microbe interactions in dietary and drug responses of *C. elegans* (Watson *et al*, 2014; García González *et al*, 2017; Rosener *et al*, 2020).

The titratable TF library introduced here is designed as a readily available genome‐wide tool that enables quantitative control of individual TFs in *E. coli* (Fig 1A). Classic techniques for studying TF function rely on completely knocking out or over‐expressing a given TF in order to infer the regulatory or physiology role. The resource introduced here provides the ability to control a TF in order to observe its role as a function of its concentration, which is measurable thanks to a fluorescent fusion to the TF. Circumventing natural TF copy number control is particularly important since TF genes are typically auto‐regulating, which makes TF control difficult; here the TF is expressed entirely from a synthetic promoter that can be induced with the small molecule aTC.

Many established tools, such as ChIP‐seq and SELEX, have been developed for the purpose of determining where TFs bind and to which sequences they prefer to bind. Our library is designed to be a complementary tool aimed at aiding studies seeking to determine a TFs regulatory function once bound (Fig 1A). The titratable TF library is versatile and can be combined with other single‐gene mutant library collections such as the transcriptional reporter library (Zaslaver *et al*, 2006), the keio single‐gene deletion library (Baba *et al*, 2006), or the isolated regulatory position sweep library developed in our lab previously (preprint: Guharajan *et al*, 2021) in order to make controlled measurements of TF regulation. The ability to isolate and control the expression of individual TFs allows for characterization of the role of individual TFs without entanglement from their physiological function or role in higher‐order network effects such as autoregulation (Potvin‐Trottier *et al*, 2016; Ali *et al*, 2020). Similar strategies have previously enabled thorough characterization of individual TFs (Garcia & Phillips, 2011; Brewster *et al*, 2014), our library enables these approaches on a TF‐wide level (Fig 1A).

We have demonstrated this use with two case studies examining regulation by the zinc‐responsive TF, ZntR, and the paralogous TFs, GalR and GalS. In both cases, we were able to isolate and quantify TF function through the use of the appropriate TF titration library strains. Another crucial tool in both of these studies is the presence of a quantitative model to interpret the data; the TF titration library enables the characterization of gene regulation in a simplified system where TF number is under control, which, in turn, enables simpler models with fewer parameters that are not intrinsic to the TF itself. For instance, in both examples, we measured regulation of the studied TFs immediately downstream of the promoter as a way to infer the affinity of our TFs to a binding site, in one case this demonstrated that the binding affinity of ZntR is independent of zinc concentration. Crucially for this case, knowing the affinity is not enough to predict regulation, you must also know the regulatory effect of the TF once bound. This is where our methodology of TF characterization shines.

In the other example presented here, we measure regulation for TF‐binding downstream of the promoter, which demonstrated the differences in binding affinity of GalR and GalS to specific binding sequences. This is a crucial step towards disentangling affinity from function. As a result, we could quantify how the TF function of the two paralogs differed in terms of not only their affinity for specific sequences but also in their function when bound to those sequences.

Immense progress has been made in genetic circuit design using TFs from a single family of TFs (Nielsen *et al*, 2016; Chen *et al*, 2020). Overall, the goal of improving the repository of well‐characterized TFs to include a greater diversity and more orthogonality has the potential to enable genetic circuit design of more complex responses with overall simpler architectures with fewer parts. Ideally, this library can be used to not only gain an understanding of natural regulation but to also improve our understanding of the general input‐output function of TF regulation and expand the toolkit of synthetic biology (Fig 1A).

## Materials and Methods

### Construction of TF library

*Escherichia coli* MG1655 is the parent strain used in our library construction. Single‐gene deletions of TF genes in the Keio library (with BW25113 as parent strain) are moved by P1 transduction into *E. coli* MG1655 expressing constitutive TetR at the *gspI* locus, and the kanamycin cassette associated with the keio knockout is flipped using the *frt* flippase expressed from the pCP20 plasmid. These strains serve as no fluorescence (and no TF) control strains for the corresponding titratable TF strain in our library. Unless otherwise stated, all steps are performed in 96‐well plates. Primers to amplify TFs are designed using customized matlab codes. Individual TF genes are cloned by Gibson assembly into pSC101 plasmid between the *P*_tet_ and aek‐linker‐mCherry sequence. Gibson clones were confirmed by sequencing and used as a template to amplify the *P*_tet_‐tf‐aek‐mCherry fusion gene for integration at the *ybcN* locus. Chromosomal integration is assisted by the lambda red recombinase proteins (*exo*, *beta*, and *gamma*) expressed from pKM208 (Murphy & Campellone, 2003) in wild‐type MG1655. Successful integrants were confirmed by sequencing and moved by P1 transduction into the corresponding TF control strains. Initial versions of the library are constructed with the mCherry sequence (here then referred to as *mCherry^wt^*) that was later reported to have an internal start codon (preprint: Fages‐Lartaud *et al*, 2021). Having such an aberrant isoform might interfere with the accurate quantification of the TF copy number. Hence, the mCherry sequence of the original library was systematically modified to have a substitution of amino acid, methionine (ATG) at position 10 with a leucine (CTG). Unless otherwise specified, all mCherry measurements are based on the mCherry variant, M10L (Refer to Appendix Figs S1A, and S4B and C) for the comparison of mCherry fluorescence from *mCherry^wt^* and *mCherry^M10L^*. Expression of additional cognate genes might be needed for TFs requiring modifications (such as the phosphorylated TFs, Appendix Fig S6).

### Growth characteristics

For each experiment, 10 different TFs from the library (no TF control and the corresponding titratable TF strains) and wild‐type MG1655 are grown in 96‐well plates to characterize the growth and mCherry fluorescence in M9‐minimal media supplemented with glucose. The strains are grown overnight in LB and diluted 10^4^‐fold into M9‐minimal media with different aTC concentrations (0, 1, 3, 5, 7, 9, and 15 ng/ml) and grown in 2 ml volume 96‐well plates at 37°C and 250 rpm until it reaches OD600 of 0.4. Ten microlitres of these cells are then diluted into 190 µl of the same media in 300 µl volume 96‐well plates and transferred to a plate reader with an automated setting (TECAN MPro‐200). OD600 values and mCherry fluorescence are measured every 30 min for up to 20 h. The growth rate is then calculated in the exponential phase of growth. Background subtracted OD600 values are log‐transformed and a polynomial fit is performed over a sliding window. A plot of the fit values across the sliding window gives characteristic regimes: a noisy regime for the lag phase, a distinct peak in the growth phase, and a plateau for the stationary phase. The maximum of the peak value corresponds to the growth rate of the particular strain. The growth rates of the test strains are normalized by the growth rate of MG1655 (grown in the same aTC concentration as the test strains) measured on the same day. Normalized growth rates are used in hierarchical clustering to determine different characteristic features of TF titration on growth rates.

### Estimation of calibration factor

The calibration factor, *v*, for the conversion of mCherry fluorescence to TF copy number is quantified as described in Brewster *et al* (2012), by measuring the stochastic fluctuations in fluorescence partitioning during cell division. Briefly, cells expressing the TF‐mCherry fusion protein are grown as described in the section for Microscopy, and just before imaging 100 µl of cells from different aTC concentrations are pooled together and washed twice with M9‐glucose minimal media containing no aTC to stop any further production of mCherry. Cells are then spotted on 2% low melting agarose pad made with M9‐glucose minimal media. All samples are imaged on an automated fluorescent microscope (Nikon TI‐E) with a heating chamber set to 37°C overnight. Phase images are captured for roughly 100 fields and their positions are saved for later. These phase images (named as Lineage tracker) will serve as a source file for lineage tracking of the daughter cell pairs (*I*_1_ and *I*_2_). After one doubling time (roughly 1 h or depending on the doubling time for different TFs), the microscope stage was returned to the same field of view using the saved position matrix and is imaged again (and named as daughter finder) now using both phase and mCherry channels. The exposure time for mCherry channel was set to 1 s and all nine TFs were measured simultaneously with identical experimental settings. For partition statistics to estimate the calibration factor cells saved as daughter finder are segmented using a modified version of Schnitzcells code (Rosenfeld *et al*, 2005). Daughter pairs (*I*_1_ and *I*_2_) are picked manually by matching the segmented daughter finder with the phase image in the lineage tracker. The mean pixel intensity and area of the daughter pairs are obtained using region props. The background fluorescence is estimated as described in Ali *et al* (2020) using the inverse mask of individual frames. The sum and squared difference in fluorescence are estimated from the total fluorescence of the daughter cells after division. The resulting single‐cell measurements are binned for summed fluorescence values and fitted with a binomial distribution function to obtain the calibration factor *v* for nine different TFs used in this study. Only TFs that do not follow a volumetric partitioning during division can be counted by this technique and to do so the TFs are assumed to be distributed randomly on the chromosome. Clearly as shown in Fig 1E, there are TFs (such as DhaR and NrdR) that are localized in the cell and might not follow random partitioning during division. Such TFs cannot be counted by this technique. In addition, calibration factors for TFs that require co‐factor or other modifications (Appendix Fig S2B and C) might be challenging to measure in this manner.

### Estimation of absolute TF numbers for the entire library

For mCherry measurements, titratable library strains are grown overnight in LB and diluted 10^4^‐fold into M9‐minimal media with different aTC concentrations (0, 1, 3, and 7 ng/ml) and grown in 2 ml volume 96‐well plates at 37°C and 250 rpm until it reaches OD600 of 0.2–0.4. Cells are then washed twice in 1X M9‐minimal media with no sugar and supplemented with spectinomycin to ensure cells are arrested and there is no more protein synthesis. Cells are then spotted on 2% agarose bed made with 1X‐M9‐minimal media with no sugar (and with spectinomycin) and imaged using conditions identical to that for calibration factor measurements described above. Six beds are imaged per sample in both phase and mCherry channel. Phase images are segmented using a modified version of Schnitzcells code (Rosenfeld *et al*, 2005). Mean pixel intensity of the mCherry fluorescence and pixel area of the cell is obtained using region props, an inbuilt function in matlab. Background fluorescence is calculated using wild‐type strain MG1655. Total fluorescence is calculated by multiplying the background‐subtracted mean pixel intensity with the total pixel area of the cell. mCherry fluorescence is further converted to protein copy number per cell by dividing by the mean of the calibration factor *v* (as estimated above for nine TFs). Each data point is a mean of single‐cell mCherry values of the given TF grown at the specified aTC concentration. Error bars are the standard error in single‐cell fluorescence. Roughly, 150–1,000 cells are analyzed per sample. The data from Schmidt *et al* (2016) are used to compare the TF copies to the physiological concentration. The growth and culture conditions between our work and the work of Schmidt *et al* (2016) have few variations, which might cause discrepancy in the actual physiological concentration of TFs. We grow cells in 200 µl volume in a 96‐well plate shaking at 250 rpm whereas in the work of Schmidt *et al* (2016) cells were grown in 50 ml volume in 500 ml baffled flasks shaking at 300 rpm. In addition, the M9 media used in this study does not have any supplements (other than the carbon source) whereas in Schmidt *et al* (2016) the media is supplemented with trace elements and thiamine.

### Microscopy and data analysis

ZntR knockout strain (no TF control strain) is used as an autofluorescent strain for microscopic measurement and the same strain transformed with the corresponding reporter plasmid serves as the constitutive strain. The ZntR‐TF‐titration strain from the library is directly transformed with different ZntR reporter plasmids. For GalR‐GalS reporter assays, the autofluorescence control strain is a double knockout of *galR* and *galS* and the constitutive strain is the double knockout strain transformed with the reporter plasmid. The titratable‐TF‐mCherry fusion construct for GalR and GalS from the library strain collection is P1‐transduced into the double knockout control strain to make the corresponding titratable library strains. These strains are then transformed with the corresponding reporter plasmids for binding assay. *mCherry^wt^* variant is expressed in strains used for the measurements related to GalR and GalS. The difference in TF copy number accounted for by the internal isoform in *mCherry^wt^* is corrected using the linear correlation between *mCherry^wt^* and *mCherry^M10L^* variants as shown in Appendix Fig S4B and C.

For microscopic analysis, control strains and their respective constitutive and titratable strains are grown overnight in LB media and diluted 10^5^‐fold into fresh M9‐minimal media supplemented with glucose and different concentration of aTC. These strains are grown for 16–18 h until it reaches a OD600 of 0.2–0.3. For experiments with zinc as a co‐factor, 0.1 M stock of fresh zinc sulfate is added to the media at a concentration of 125 µM and serially diluted to the desired zinc concentrations. As zinc sulfate is volatile the stocks are stored at −20°C and thawed right before use. We observe significant degradation of zinc even when stored at −20°C and hence comparisons are usually made for experiments performed with the same batch of zinc. Unless otherwise specified, the aTC concentration used for all microscopic experiments are 0, 0.25, 0.5, 0.75, 1, 1.25, 1.5, 1.75, 2, 2.5, 3 ng/ml and 300 µl cells are grown in 2 ml‐deep well 96‐well plate. Once in steady state, cells from different aTC concentrations are pooled and washed twice with 1X PBS and spotted on 2% low melting agarose pad made with 1X PBS. The constitutive strains and autofluorescent strains are processed the same way as the pooled library strain and all samples are imaged on an automated fluorescent microscope (Nikon TI‐E) with a heating chamber set at 37°C. For constitutive and autofluorescence sample, 15 unique fields of view are imaged resulting in roughly 100–300 cells per sample. For pooled titration strains, 40 unique fields are imaged per sample resulting in 1,000–2,000 cells per sample.

Segmentation of individual cells is performed using a modified version of the matlab code, Schnitzcells (Rosenfeld *et al*, 2005). We use this code to segment the phase images of each sample to identify single cells. Mean pixel intensities of YFP and mCherry signals are extracted from the segmented phase mask for each cell using region props, an inbuilt function in matlab. The autofluorescence is calculated by averaging the mean intensity of the autofluorescence strain in both mCherry and YFP channels and is subtracted from each measured YFP or mCherry value. Total fluorescence for each channel is obtained by multiplying the mean pixel intensity with the area of the cell. Fold change in expression for a given binding site is calculated by the ratio of total fluorescence of strains expressing the TF to the strains with no TF. Fold change is also calculated for the TF strains to the knockout expressing target YFP constitutively, without any regulation by the TF. The final fold change is the ratio of fold change with regulation and without the regulation. mCherry values are converted to TF numbers using the measured calibration factor for each individual strain. The values are binned for TF number. For each experiment, three independent measurements are made and binned for TF number. Each data point is the mean and standard deviation of the binned value of each independent experiment.

### Kinetic modeling

Based on the observation in Fig 4D, we built a simple kinetic model to explore the regulatory features of ZntR (TF) mediated regulation of a promoter that drives the expression of ZntA transporter (E). In the model, external zinc (*Zinc*_out_) is transported inside the cell with a rate *β*_in_ whereas the exporter facilitates the export of internal zinc (*Zinc*_in_) outside the cell with a rate *β*_out_. We assume that the TFs inside the cell can either be zinc bound (TF*) or free (TF). The basal expression from a TF‐free promoter (*P*_off_) and a TF*‐bound promoter ($P_{\text{on}}^{*}$) are *r*_0_ and *r*, respectively. We further assume that the free TF‐bound promoter (*P*_off_) completely represses the promoter. Here, by modulating the values of *r* with respect to the basal expression rate *r*_0_, we can study the effect of different promoter strengths, e.g., for a saturating zinc concentration, a promoter with *r* > *r*_0_ will act as an activator whereas a promoter with *r* < *r*_0_ will act as a repressor. The set of ordinary differential equations (ODEs) describing the dynamics of the system is given below.

(2) $$\begin{array}{c} \frac{d[Zinc_{\text{in}}]}{\mathrm{dt}}=\beta_{\text{in}}\left[Zinc_{\text{out}}\right]‐\beta_{\text{out}}\left[Zinc_{\text{in}}\right]\left[E\right]‐\gamma_{1}\left[Zinc_{\text{in}}\right]\left[\text{TF}\right]+\gamma_{2}\left[{\text{TF}}^{*}\right],\\ \frac{d[{\text{TF}}^{*}]}{\mathrm{dt}}=\gamma_{1}\left[Zinc_{\text{in}}\right]\left[\text{TF}\right]‐\gamma_{2}\left[{\text{TF}}^{*}\right]‐k_{\text{on}}\left[{\text{TF}}^{*}\right]\left[P_{\text{off}}\right]+k_{\text{off}}\left[P_{\text{on}}^{*}\right],\\ \frac{d[P_{\text{on}}]}{\mathrm{dt}}=k_{\text{on}}\left[\text{TF}\right]\left[P_{\text{off}}\right]‐k_{\text{off}}\left[P_{\text{on}}\right],\\ \frac{d[P_{\text{on}}^{*}]}{\mathrm{dt}}=k_{\text{on}}\left[{\text{TF}}^{*}\right]\left[P_{\text{off}}\right]‐k_{\text{off}}\left[P_{\text{on}}^{*}\right],\\ \frac{d[E]}{\mathrm{dt}}=r\left[P_{\text{on}}^{*}\right]+r_{0}\left[P_{\text{off}}\right]‐\gamma \left[E\right], \end{array}$$

with the following constraints,

(3) $$\begin{array}{c} \left[P_{\text{off}}\right]+\left[P_{\text{on}}\right]+{\left[P_{\text{on}}\right]}^{*}=1,\\ {}[\text{TF}]+\left[{\text{TF}}^{*}\right]+\left[P_{\text{on}}\right]+\left[P_{\text{on}}^{*}\right]=\left[{\text{TF}}_{\text{total}}\right],\\ {}[{\text{TF}}^{*}]\;+\;\left[P_{\text{on}}^{*}\right]+\left[Zinc_{\text{in}}\right]+\left[Zinc_{\text{out}}\right]=\left[Zinc_{\text{total}}\right]. \end{array}$$

Here, *γ*_1_ and *γ*_2_ are the rates at which the TF binds and unbinds intracellular zinc. *P*_on_ and $P_{\text{on}}^{*}$ are the concentrations of promoters bound by TF and TF*. *γ* is the degradation rate of the transporter, *E*. For simplicity, the transporter (*E*) itself is the reporter gene here. In Fig 4D, we show that the fold change versus TF curves are similar, which tells us that the binding affinity (*k*_on_ and *k*_off_) of TF and TF* to the promoter is the same or equivalently, both the TF and TF* bind DNA with a rate *k*_on_ and unbind with a rate *k*_off_. The right‐hand side of the above equations is set to zero in order to obtain the steady‐state values of each component. Interestingly, the steady‐state solutions require only few free parameters, *r*, *r*_0_, and experimentally observed parameters, the ratio *k*_on_/*k*_off_ and the degradation rate *γ* = log(2)/cell cycle. In Fig 4G, we plot the fold change of the exporter (E) as a function of effective intracellular zinc concentration (*Z*_out_ *γ*_1_ *β*_in_/γ_2_ *β*_out_).

## Author contributions

**Vinuselvi Parisutham:** conceptualization, data curation, formal analysis, validation, investigation, visualization, methodology, and writing—review and editing. **Shivani Chhabra:** conceptualization, data curation, investigation, and methodology. **Md Zulfikar Ali:** software and methodology. **Robert C Brewster:** conceptualization, formal analysis, supervision, funding acquisition, investigation, writing—original draft, project administration, and writing—review and editing.

In addition to the CRediT author contributions listed above, the contributions in detail are:

RCB conceptualized the library. VP and SC performed all the experiments for library construction. VP performed all the case study. MZA helped with the model. RCB and VP developed the manuscript.

## Library distribution

The TF library strains are available for distribution, free of cost.

## Disclosure and competing interests statement

The authors declare that they have no conflict of interest.

## Supporting information



Appendix

Table EV1

Source Data for Figure 1

Source Data for Figure 2

Source Data for Figure 3

Source Data for Figure 4

Source Data for Figure 5

## Data Availability

This study includes no data deposited in external repositories. All essential data are available as Source Data. Primers used in the library construction are listed in Table EV1.
