# Supplementary material for: Tunable transcription factor library for robust quantification of regulatory properties in Escherichia coli
Source: Mol Syst Biol. 2022 Jun 13;18(6):e10843. doi: 10.15252/msb.202110843 (PMC9189660; doi:10.15252/msb.202110843)
Supplement: Supplementary file 1 — Appendix [file MSB-18-e10843-s002.pdf]

Tunable Transcription Factor Library for Robust Quantification of Regulatory Properties in *E. coli*

Vinuselvi Parisutham[1], Shivani Chhabra[3], Md Zulfikar Ali[1], Robert C. Brewster[1,2,*]

May 11, 2022

1. Department of Systems Biology, University of Massachusetts Chan Medical School, 368 Plantation St., Worcester, MA 01605.
2. Department of Microbiology and Physiological Systems, University of Massachusetts Chan Medical School, 368 Plantation St., Worcester, MA 01605.
3. Department of Pharmacological Sciences, Icahn School of Medicine at Mount Sinai, New York, NY 10029.

* Corresponding Author: Robert C. Brewster, Robert.Brewster@umassmed.edu.

Subject categories: Molecular microbiology

Keywords: Transcription regulation, quantitative gene regulation, bacterial physiology, genetic library, paralogs.

Running Title: Tunable transcription factors

Contents

Appendix Figure S1: Systematic swapping of the fluorophore

Appendix Figure S2: Transcription factor library

Appendix Table S1: Kinetic parameters used in the model

Appendix Figure S3: Regulation by ZntR

Appendix Figure S4: Isorepressors - GalR/GalS

Appendix Figure S5: Measurements of calibration factor

Appendix Figure S6: Phosphorylated transcription factors

Appendix Figure S7: Global regulator H-NS

Appendix Figure S8: Individual clusters of the clustergram in Fig 3A

Systematic swapping of the fluorophore

Following the footsteps of our previous studies with LacI (Brewster *et al*, 2014), CpxR and few other TFs (Guharajan *et al*, 2021), we used mCherry to tag the TFs in our library. Choice of mCherry also makes our library compatible with the GFP and YFP reporters in Zaslaver's transcription reporter library (Zaslaver *et al*, 2006) and our lab's binding site position sweep library (Guharajan *et al*, 2021), respectively. However, we do not expect that mCherry will serve as an universally compatible tag for all the TFs in our library and there would be cases where mCherry could interfere with the functionality of the TF in unprecedented ways. Previously, different fluorescent proteins where tested for their ability to form foci and mCherry was one of the several fluorescent proteins that might form aberrant fluorescent foci (Landgraf *et al*, 2012). In addition, recent reports indicate mCherry has an alternate initiation site 10 amino acid downstream of the actual start site (Fages-Lartaud *et al*, 2021). We tested the interference of this alternate initiation site in our library strain and realized that a significant fraction of the signal arise from the alternate start codon. In our library, the TFs are integrated in a common genetic loci (*ybcN*) with a linker sequence separating the TF and the mCherry and hence it is relatively easy to make any necessary alterations in a systematic way using just a pair of primer. For instance, we can replace mCherry with a different fluorophore by exploiting the AEK linker (36 bases long) as the upstream homologous region or we can replace just one nucleotide of the linker (replacing the 19th nucleotide (G) in the linker with a T) to introduce a stop codon and express un-tagged TFs inside the cell or even systematically add bar-codes to be used in several high-throughput experiments.

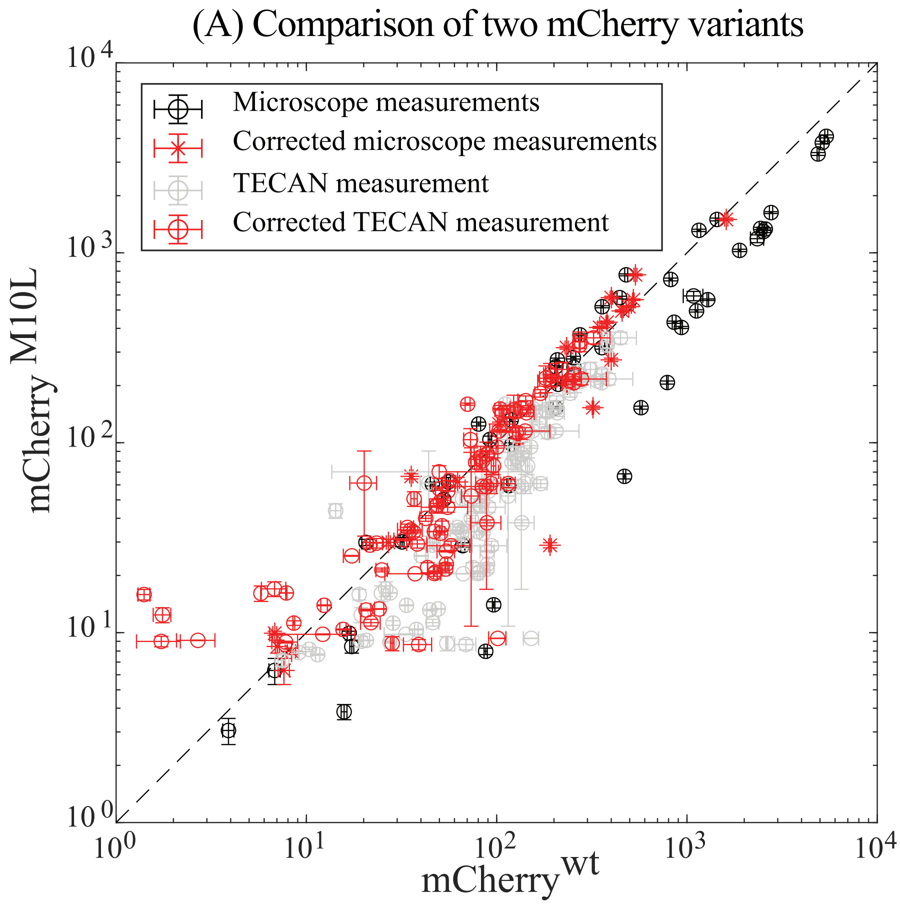

Appendix Figure S1: mCherry variants (A) Comparison of TF copy number measured in strains expressing $mCherry^{wt}$ and $mCherry^{M10L}$ variants.

Transcription factor library

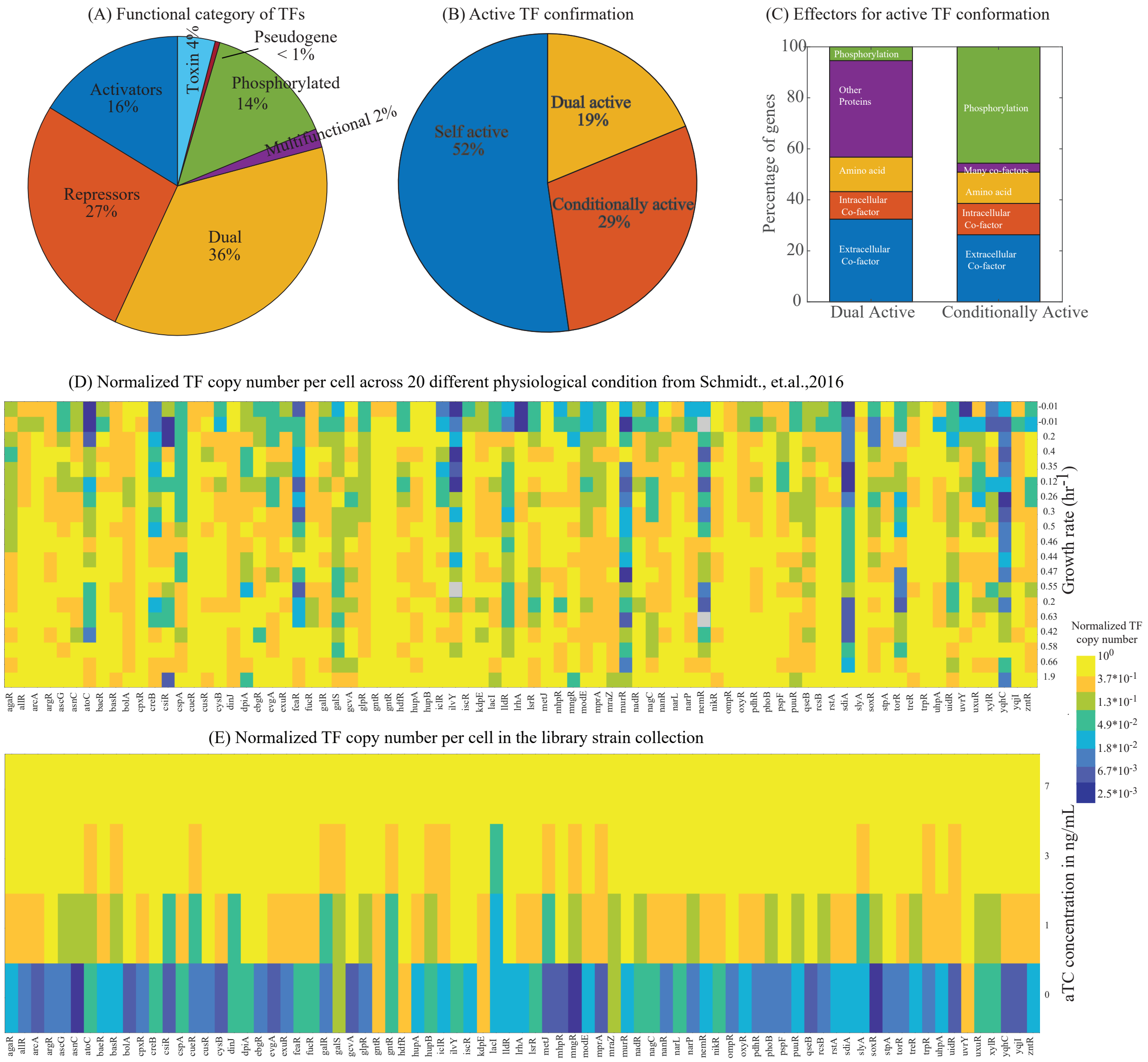

Appendix Figure S2: Transcription factor library: **(A)** Based on the mode of action, TFs can be classified into seven functional categories: Activators, repressors, dual regulators, toxin-antitoxins, phosphorylated TFs, pseudogenes and multi-functional TFs. **(B)** TFs can be classified into 3 categories based on the conformation with which they bind the DNA *i.e.* active TF conformation. **(C)** The effector molecules used by TFs to facilitate DNA binding or active TF conformation. **(D)** Physiological concentration of different TFs (normalized to the average TF obtained in the library strain at maximum induction) in wild type strains as measured by Schmidt *et. al.* (Schmidt *et al*, 2016). As shown in this figure, altering the growth rate is not a good proxy to achieve different levels of TFs due to the inherent regulations of the native TF. **(E)** Every TF in our library strain is titratable to 100-1000s of TF copy number under identical induction condition (normalized to the average TF obtained in the library strain at maximum induction).

Kinetic parameters used in the model

Appendix Table S1: Kinetic rates used in the simulations for Fig 4F

Rates	Symbols	Value
Degradation rate	γ	$0.0002\ \mathrm{s}^{-1}$
Binding energy (Experimentally verified)	$k_{\mathrm{on}}/k_{\mathrm{off}} = exp(-\Delta\epsilon)/N_{\mathrm{ns}}$	0.2
Rate of basal expression	r_0	$0.002\mathrm{s}^{-1}$ $0.0760\mathrm{s}^{-1}$
Rate of activation	r	$0.02\ \mathrm{s}^{-1}$
Effective zinc concentration	$Zinc_{\mathrm{out}}(\gamma_1/\gamma_2)(\beta_{\mathrm{in}}/\beta_{\mathrm{out}})$	$0.01 - 2000\mu M$

Regulation by ZntR

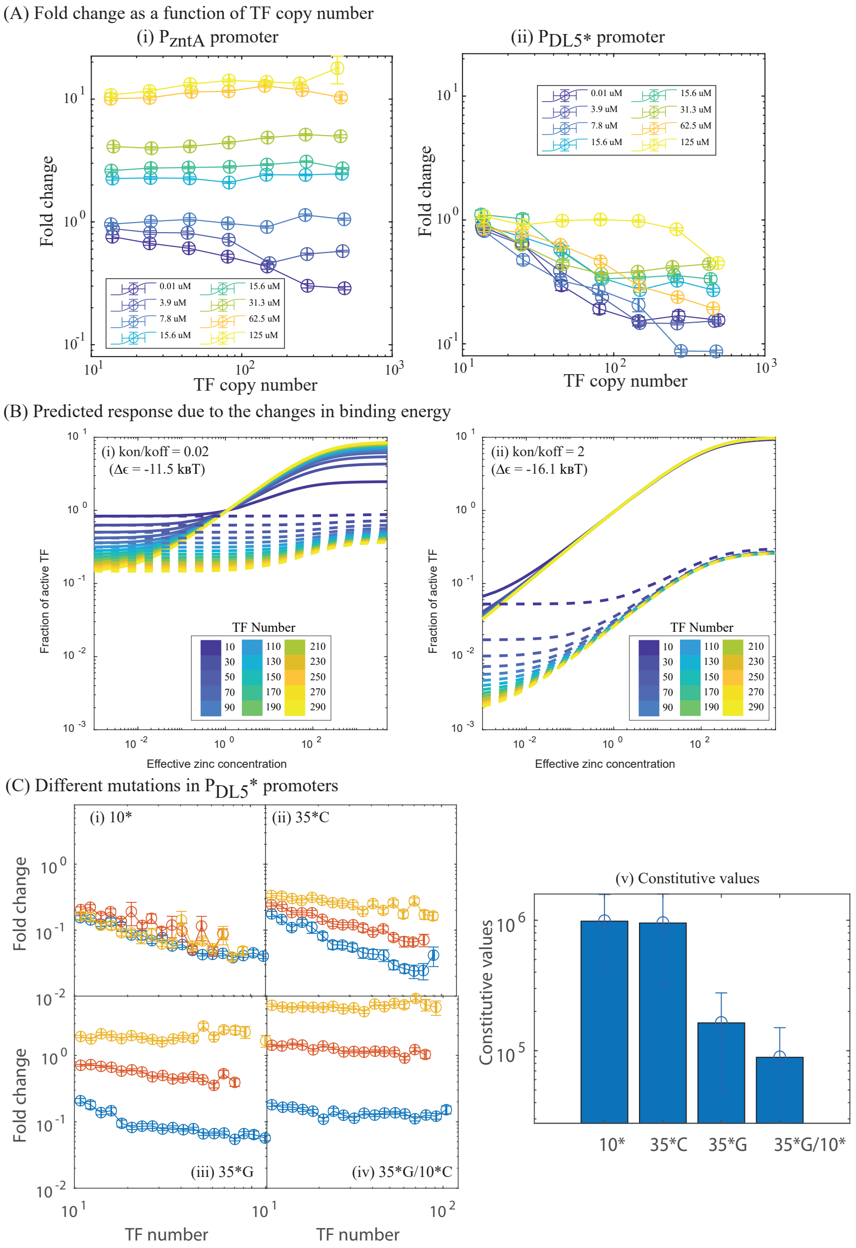

Appendix Figure S3: ZntR and zinc regulation: (A) Data from Fig 4C plotted as a function of TF-copy number for varying zinc in (i) P_{zntA} promoter and (ii) P_{DL5^*} promoter. **(B)** Predicted response from the kinetic model for different TF binding affinities $k_{\text{on}}/k_{\text{off}}$. The affinity is made weaker (i) or stronger (ii) compared to the computed binding energy for ZntR in Fig 4E. **(C)** Fold change in response to zinc for different modifications in the -35 and -10 boxes of the P_{DL5^*} promoter. (i) 10^* is replacing the AA of P_{DL5^*} with the CC of P^*_{zntA}. (ii) 35^* is replacing the T at position 3 of the P_{DL5^*} with a C. (iii) 35^*G is replacing the T of P_{DL5^*} with the G of P^*_{zntA}. (iv) $35^*/10^*$ is replacing both the mutations from P^*_{zntA} to P_{DL5^*}. Ideally this construct is same as the P^*_{zntA} except for the mutations in the the untranslated region just downstream of the transcription start site (refer to Fig 4B). (v) Constitutive values from each of the construct in (i-iv).

Isorepressors - GalR/GalS

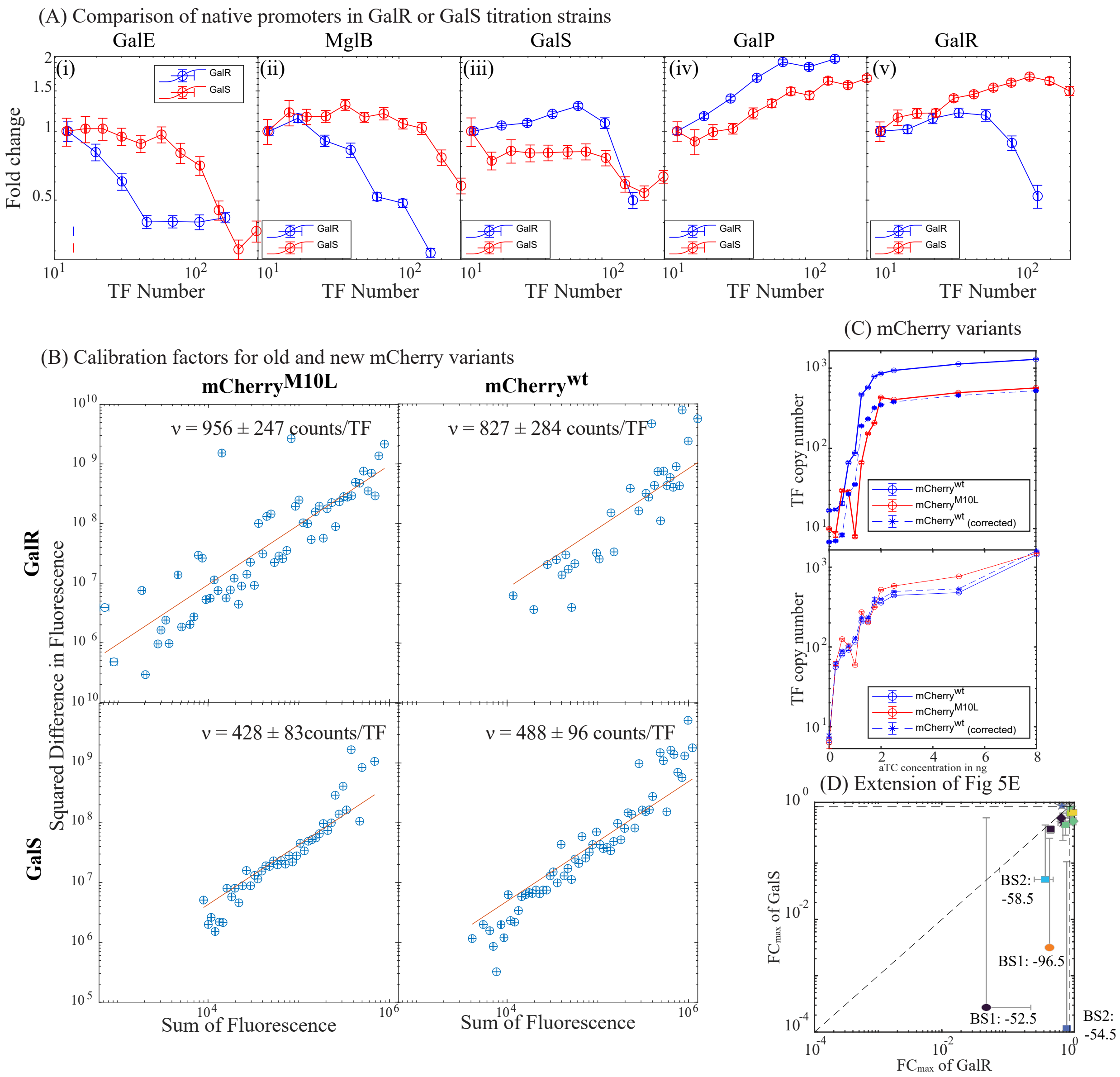

Appendix Figure S4: Isorepressors: (A) The regulatory curves for GalR and GalS on their native promoters from the Zaslaver transcriptional reporter library. Three different trials of the experiment are normalized to the corresponding fold change and binned together. Error bar represents the standard error for the corresponding bin. **(B)** Comparison of calibration factor for GalR and GalS fused to two different mCherry variants ($mCherry^{M10L}$ and $mCherry^{wt}$. Two different mCherry variants did not have huge difference in the measured calibration factor. **(C)** For experiments in Fig 5, we used $mCherry^{wt}$ variant for measurements and re normalized the TF number to correspond to $mCherry^{M10L}$ variant. The qualitative details of the experiment will not change with the different variants of mCherry. However, binding energy and other quantitative features are under-mined with $mCherry^{wt}$ variant as it over counts the TF number. **(D)** Extension of Fig 5E showing the lower quadrant for repression in both GalR and GalS.

Measurements of calibration factor

Calibration factor is a measure of stochastic event and care should be taken to avoid or minimize contributions from extrinsic factors during experimental set up and data processing. Major factors affecting the experimental set up are (1) efficient washing of cells in order to remove any traces of aTC and shut-down any further expression of mCherry, (2) incubator associated with the microscope should be equilibrated to 37° C several hours before the experiment, (3) the precision in the time to capture one division (images taken too early might result in far less cells and images taken several minutes after the first division might have volumetric difference), and (4) finally the lower signals (major contributors of the y-intercept or the calibration factor) is largely dependent on efficiency with which background signals are calculated. As we have shown previously (Ali *et al*, 2020), background fluctuations are critical especially for lower signals that are just couple of counts above the background. Hence, we use error-propagation to account for any errors that could arise from changes in background counts in estimating the sum of the fluorescence or in squared difference in fluorescence.

$$\begin{aligned} \sigma_{\text{sum}} &= (I_1 + I_2)\sigma_{\text{bg}}, \\ \sigma_{\text{diff}} &= 2(I_1 - I_2)(A_1 - A_2)\sigma_{\text{bg}}, \end{aligned} \tag{1}$$

Here, $I_1 + I_2$ are the fluorescence intensity of mother-daughter pair and σ_{sum}, and σ_{diff} are the standard deviation in the measure of sum and difference in fluorescence between the daughter cells and σ_{bg} is the standard deviation of the auto-fluorescent strain. σ_{bg} is measured experimentally. σ_{sum}, and σ_{diff} are inferred from the above relationship. A σ_{bg} value of up to 5 is tolerable in the measurements of calibration factor.

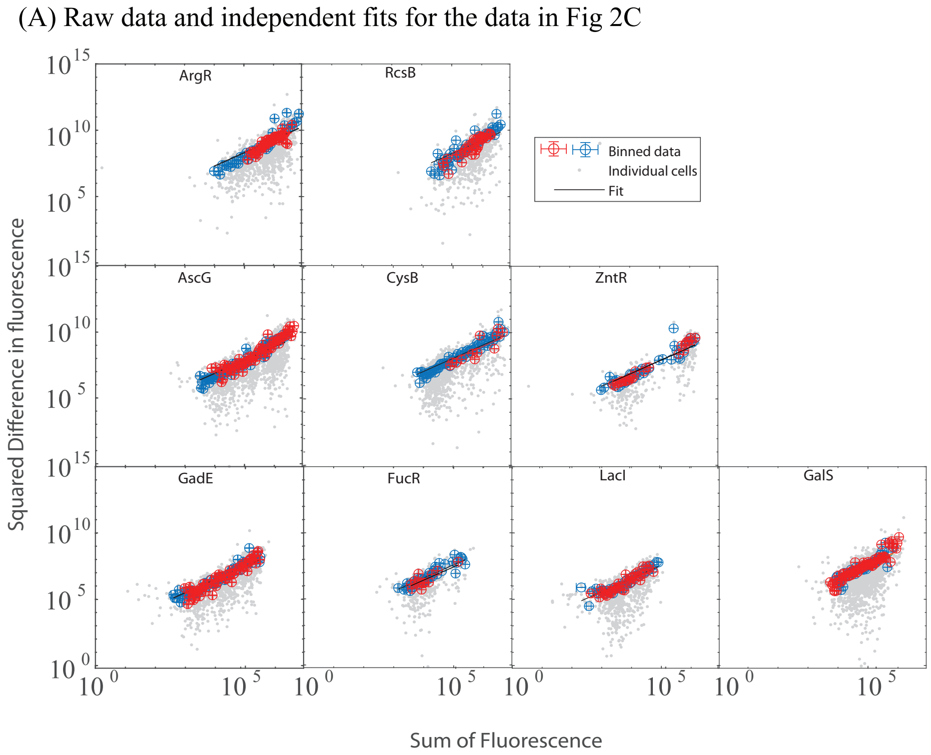

Appendix Figure S5: Independent calibration factor curves (A).Plot showing the independent calibration curves for the 9 different TFs. The plots are grouped according to their calibration value. As a general trend we observe a correlation between the distribution of signal and the values for the calibration factor.

Phosphorylated transcription factors

Response regulators of the two component signal transduction system of *E. coli* are TFs that require phosphorylation and there are a total of 26 of them in our library strain collection. These TFs present additional challenges in quantitative studies as these proteins take up distinct regulatory roles proportional to its phosphorylation status inside the cell. Discrete protein kinase, usually encoded along side the associated response regulator (or the TF) is essential for phosphorylation of the corresponding TF. However, in our library strain, only the response regulators' concentration is controlled and not the concentration of kinase. In addition, the kinase is usually encoded within the same operon expressing the response regulator and in our library strains the response regulators are deleted in-frame from its native locus. Such deletions could easily alter the expression of the kinase associated in the same operon. Hence, phosphorylated TFs might need additional genetic modifications such as deletion or regulated expression of the regulated kinase or replacing the critical amino acids of response regulator with phospho-mimetic amino acids (Schastnaya *et al*, 2021) or choice of growth characteristics that would naturally induce phosphorylation. Although we have succeeded characterizing the phosphorylated TF, CpxR (Guharajan *et al*, 2021) in the past, for most other TFs phosphorylation remains challenging as in Fig S6. CpxR might be special case as it activates the expression of its own kinase but may not be generalized for other kinase and need careful characterization.

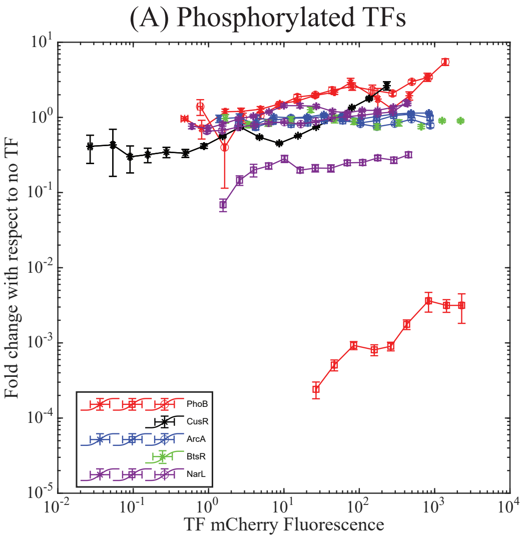

Appendix Figure S6: Input-output relationship for selected phosphorylated TFs (A) Input-output relationship is measured for TFs: PhoB, CusR, ArcA, BtsR and NarL with selected plasmids from Zaslaver's transcriptional reporters.The promoters for PhoB are P_{phnC} (red star), P_{phoB} (red square), and P_{phoH} (red circle). Auto-regulatory promoter P_{cusR} is used for CusR library strain. The promoters for ArcA are P_{icd} (blue star), P_{ssb} (blue square), and P_{uvrA} (blue circle). The promoter for BtsR is P_{yjiY} (green circle). The promoters for NarL are P_{aspA} (purple star), P_{dcuS} (purple square), and P_{focA} (purple circle). These group of proteins require phosphorylation for its response regulation. $mCherry^{wt}$ variant is used in these experiments.

Global regulator H-NS

Global regulator, H-NS is one of those few TFs whose predicted TF concentration is lower than the reported physiological concentration (Fig 2E). About 5% of genes in *E. coli* is regulated by H-NS (Hommais *et al*, 2001). Hence, we tested the physiological effect of titration of H-NS in several minimal media (Fig S7A-B). As shown in panel B. the mCherry levels are similar in different media (M9-glucose, M9- arabinose and M9-glycerol media) however, the amount of aTC required to rescue growth equivalent to the wild-type is different under the 3 tested condition with growth in glycerol requiring more H-NS proteins to rescue wild-type like growth than glucose or arabinose minimal media. This also demonstrates yet another functionality of our library strains .i.e. in studying the limitations of TF titration under different nutrient conditions.

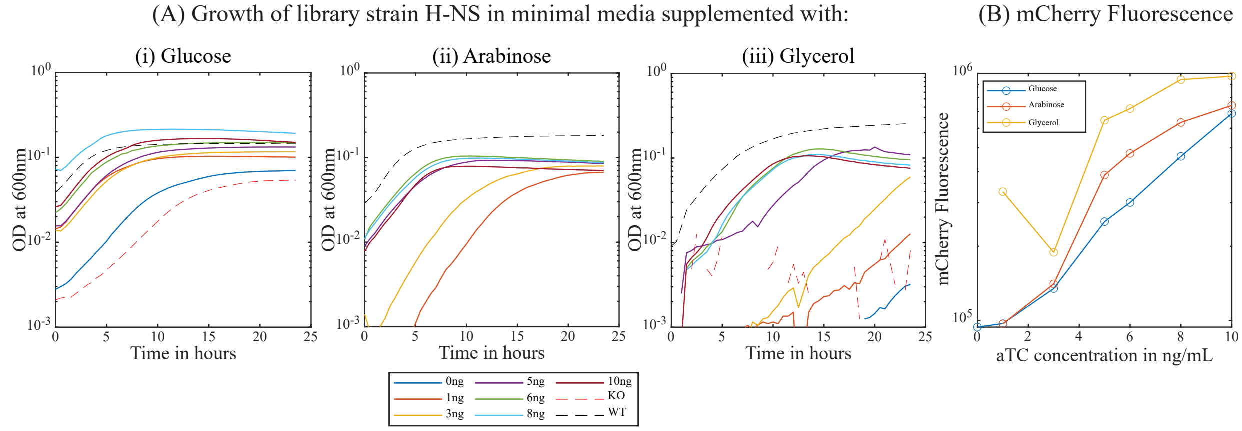

Appendix Figure S7: Global regulator H-NS (A) Plot showing the growth of H-NS knockout and titratable library strains in minimal media supplemented with (i) Glucose (ii) Arabinose or (iii) Glycerol as a sole carbon source. H-NS is a global regulator and one of those library strain's with the TF numbers lower than the physiological concentration. **(B)** Plot of the mCherry levels as a function of aTC concentration in different growth medias for the library strain, H-NS.$mCherry^{wt}$ variant is used in these experiments.

Individual clusters of the clustergram in Fig 3A.

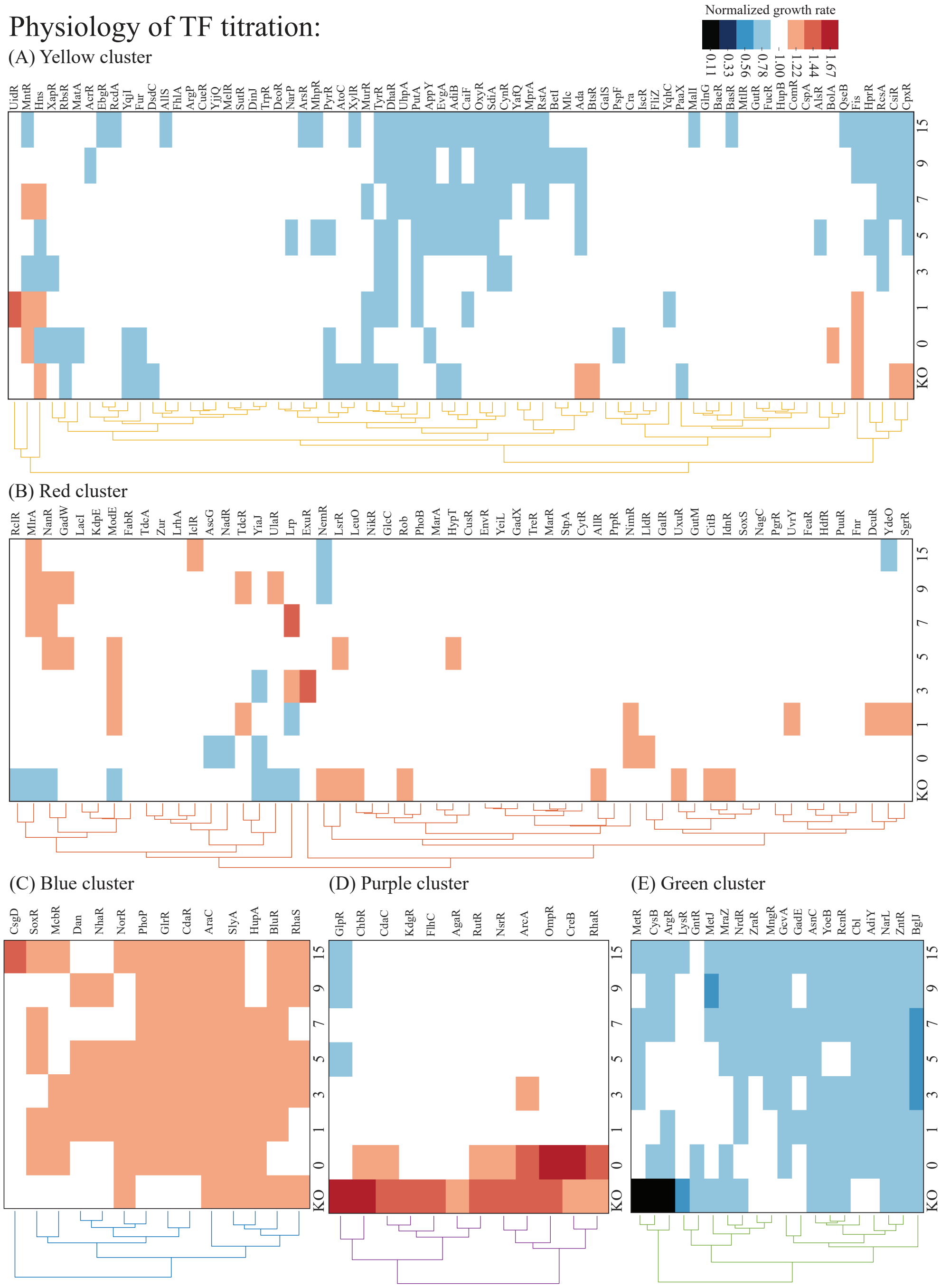

Appendix Figure S8: Individual clusters: Data in Fig 3A with 5 individual clusters presented separately and gene names displayed. The last cluster in Fig 3A includes only 2 TFs (Nac and PdhR) and is not shown here.

References

Ali MZ, Parisutham V, Choubey S, Brewster RC (2020) Inherent regulatory asymmetry emanating from network architecture in a prevalent autoregulatory motif. *Elife* **9**

Brewster RC, Weinert FM, Garcia HG, Song D, Rydenfelt M, Phillips R (2014) The transcription factor titration effect dictates level of gene expression. *Cell* **156**: 1312–1323

Fages-Lartaud M, Tietze L, Elie F, Lale R, Hohmann-Marriott MF (2021) mCherry contains a fluorescent protein isoform that interferes with its reporter function. *bioRxiv*

Guharajan S, Chhabra S, Parisutham V, Brewster RC (2021) Quantifying the regulatory role of individual transcription factors in *Escherichia coli*. *bioRxiv*

Hommais F, Krin E, Laurent-Winter C, Soutourina O, Malpertuy A, Le Caer JP, Danchin A, Bertin P (2001) Large-scale monitoring of pleiotropic regulation of gene expression by the prokaryotic nucleoid-associated protein, H-NS. *Mol Microbiol* **40**: 20–36

Landgraf D, Okumus B, Chien P, Baker TA, Paulsson J (2012) Segregation of molecules at cell division reveals native protein localization. *Nat Methods* **9**: 480–482

Schastnaya E, Raguz Nakic Z, Gruber CH, Doubleday PF, Krishnan A, Johns NI, Park J, Wang HH, Sauer U (2021) Extensive regulation of enzyme activity by phosphorylation in *Escherichia coli*. *Nat Commun* **12**: 5650

Schmidt A, Kochanowski K, Vedelaar S, Ahrne E, Volkmer B, Callipo L, Knoops K, Bauer M, Aebersold R, Heinemann M (2016) The quantitative and condition-dependent *Escherichia coli* proteome. *Nat Biotechnol* **34**: 104–110

Zaslaver A, Bren A, Ronen M, Itzkovitz S, Kikoin I, Shavit S, Liebermeister W, Surette MG, Alon U (2006) A comprehensive library of fluorescent transcriptional reporters for *Escherichia coli*. *Nature Methods* **3**: 623–628
