# Supplementary material for: Tunable transcription factor library for robust quantification of regulatory properties in Escherichia coli
Source: Mol Syst Biol. 2022 Jun 13;18(6):e10843. doi: 10.15252/msb.202110843 (PMC9189660; doi:10.15252/msb.202110843)
Supplement: Supplementary file 8 [file MSB-18-e10843-s004.pdf]

molecular systems biology

Tunable Transcription Factor Library for Robust Quantification of Regulatory Properties in E. coli

Vinuselvi Parisutham, Shivani Chhabra, Md. Zulfikar Ali, and Robert Brewster
DOI: 10.15252/msb.202110843

Corresponding author(s): Robert Brewster (Robert.Brewster@umassmed.edu)

Review Timeline:

Submission Date:	30th Nov 21
Editorial Decision:	7th Jan 22
Revision Received:	27th Apr 22
Editorial Decision:	9th May 22
Revision Received:	11th May 22
Accepted:	13th May 22

Editor: Maria Polychronidou

Transaction Report:

(Note: With the exception of the correction of typographical or spelling errors that could be a source of ambiguity, letters and reports are not edited. Depending on transfer agreements, referee reports obtained elsewhere may or may not be included in this compilation. Referee reports are anonymous unless the Referee chooses to sign their reports.)

Thank you again for submitting your work to Molecular Systems Biology. We have now heard back from the three reviewers who agreed to evaluate your study. As you will see below, the reviewers acknowledge that the study is a relevant contribution to the field. They raise however a series of concerns, which we would ask you to address in a revision.

I think that the reviewers' recommendations are clear and therefore there is no need to repeat the points listed below. All issues raised by the reviewers need to be satisfactorily addressed. Please contact me in case you would like to discuss in further detail any of the issues raised.

On a more editorial level, we would ask you to address the following points:

Reviewer #1:

Predicting the regulatory function of a transcription factor (TF) is challenging. Although activity is influenced by concentration,

several other factors, including co-factors like small molecules or metals, degree of phosphorylation, associated proteins, and the interconnected nature of regulatory networks, where network motifs like feedforward loops are common, can also play a role. To investigate the influence of a TF's concentration in a manner that limits the influence of these confounding factors, Parisutham et al. have generated a library of close to 200 E. coli strains producing titrable TFs, where TF concentration can be manipulated by addition of a chosen amount of inducer (tetracycline). This set-up allows to study the regulatory function of a TF in a way that makes it more independent of the network properties. Furthermore, TF levels can be measured by recording the fluorescence emitted by mCherry which is fused to the TF of interest. They present two practical examples in support of their approach. The first example deals with a promoter whose activity is dependent on a TF that binds a heavy metal. In this class of promoters, the spacing between the -10 and -35 binding sequences for the sigma factor of the RNA polymerase (RNAP) is not optimal. A metal associates with a TF that is already bound between the -10 and -35 sequences, thus re-orienting the DNA in a way that will now allow the binding of the RNAP holoenzyme. In the experimental set-up, a reporter is used to measure gene activity and the concentrations of both the TF and the metal co-factor can be controlled directly by the experimenter. This strategy was successful in unveiling a formerly unknown aspect of the regulatory process, where increasing amounts of metal lead to a shift in the TF function, from repressor to activator. This subtle behavior would have been difficult to observe with conventional approaches. Unexpectedly, this approach also revealed that this transition was mostly independent from TF concentration. In the second example, the authors explore the case of paralogous TFs, i.e., those that arose from gene duplication and often recognize similar binding sequences. These TFs frequently have overlapping regulons and it is unclear if they do in fact act differently on their shared targets or if they have independent target genes. Importantly, the experimental strategy allows the independent titration of two paralogous TFs and these questions can be investigated in a controlled manner. Specifically, by studying the paralogs GalR and GalS, which bind to similar sequences, it was possible to uncover differential regulation, where one TF acts more frequently as an activator and the other as a repressor. In general, the data support the authors 'claim that their library is going to be a useful resource for studies of mechanisms of transcriptional regulation and the examples are well chosen. I only have a few comments.
Specific points:
1)Although the addition of mCherry is useful to quantify the amount of TF produced in the induction system, it is important to emphasize that the addition of mCherry may change the properties of the TF, e.g., by making it more stable or interfering with interaction with certain binding partners. It may for instance explain why the ZntR-mCherry fusion acts as a repressor in the absence of Zn. I don't think that this will limit the use of the library in most cases, but it would be helpful to comment on this possibility somewhere in the text
2)In terms of generalizing the use of the library to as many TFs as possible, it is likely that certain classes of TFs are going to pose specific problems that will need to be addressed. For instance, the activity of response regulators in two component systems is controlled by phosphorylation. Titrating the response regulator without similarly modulating the concentration of the cognate histidine kinase is unlikely to produce meaningful data. The same is true for certain TF that rely on cofactors. In the example chosen, the co-factor is a metal (Zn), whose concentration can be modified by adding specific amount to the medium. For other factors, like for cAMP or certain metabolites, it might not be as easy.
Minor:
p.6, first paragraph of discussion, letter missing, it should be "Pseudomonas aeruginosa"
p.6, last paragraph, should be "complementary tool" instead of "complimentary" unless the authors want to emphasize the fact that the use of their library is free of charge.

Reviewer #2:

Review, Parisutham et al.

Summary

In this work, the authors create a library of E. coli strains where each E. coli transcription factor has been fused to mCherry and put under the control of the Ptet promoter. In conjunction with appropriate fluorescence calibration, these strains can be used to characterize the gene regulatory function for most E. coli transcription factors under different growth conditions and for multiple gene targets.

General remarks

Characterizing the gene regulation functions for transcription factors is fundamental to obtaining a quantitative understanding of gene regulation. Even this has been achieved, at different levels of detail, for a select set of endogenous transcription factors, we lack a quantitative understanding of most transcription factors for any biological system. Tools like the one presented in this work are a valuable addition to the arsenal of methods available to scientists to study new transcription factors and better understand gene regulation.

Major comments

- It has been reported that fusions of mCherry and other fluorescent proteins can form spurious fluorescent foci (Landgraf et al.,

Nature methods, 2012). The authors encountered foci in some of their low-induction strains. Is it possible to see foci in the high-induction strains in the low concentration regime?

- What is the rationale behind using mCherry, instead of other fluorescent proteins that may show lower foci formation?

- What is the detection threshold for their method, particularly the plate reader method?

Minor comments:

- "Fourth cluster in purple" should be "The fourth cluster in purple"
- "aerugino" should be "aeruginosa"
- "Predicting the (...) is not possible". Maybe not now, but maybe in the future.
- The images are helpful and well organized, but there are multiple cases where it is tough to read the labels. Consider adjusting the font size, so it is legible when in paper format. Some examples:
• Figure 2B, inset, axis labels
• Figure 3B, Top, legend; Bottom, gene names
• Figure S1D-H, multiple cases
- Could not find references in the main text for Figure S6 or S7.

Reviewer #3:

In "Tunable Transcription Factor Library for Robust Quantification of Regulatory Properties in E. coli", the authors present a library of E. coli cell strains that enable precise control of TF concentrations. In each library strain, the authors knockout one TF gene and introduce a titratable TF copy into the genome. The TF abundances are determined using the mCherry fluorescence reporter. The authors show that this newly-generated library can be combined with rationally designed promoters to uncover the regulatory functions of individual TFs in two case studies.

This work could have a large impact on the field. Although studies on individual model TFs have been fruitful in the past decade, response functions of most TFs in E. coli are still unexplored and the quantitative understanding of their regulatory behavior is largely hindered by the lacking of single-cell level tools. The presented library covers 194 TFs in E. coli. It could potentially serve as a standard for quantitative studies on TF functions and facilitate the genetic part design for complex functions. The authors are encouraged to include a statement about how the cell strains will be available to the community.

The manuscript is well-structured and contains a large amount of quantitative data. By fine-tuning the TF number and promoter structure, the authors provide some novel insights on the regulatory functions of Zinc-responsive TF ZntR and isorepressors GalR and GalS. However, I would like to invite the authors to address several technical issues that have undermined the strength of the current manuscript. I am also not very convinced about the authors' modeling approach. Detailed comments are given below.

Major comments
- Figure 2C: besides ArgR, there are still large differences in calibration factors for other TFs. A bit of text to explain the possible reasons would be helpful. The authors have claimed that the calculation of v depends heavily on experimental settings (page 3 line 11). Do they use the same experimental settings for all 10 TFs? If so, please extend the "Estimation of calibration factor" part to include more information such as the exposure time.

- Figure 2E: how the authors estimate the absolute TF number per cell is not clear enough for me. I understand that the authors could measure TF conc. using plate reader but how do they convert the TF conc. to TF absolute number per cell? Do the authors assume the cell sizes for the library strains at maximal induction are all the same? From figure 3B, the growth rates for the strain library at maximal induction are very different and there should be changes in the cell size (Klumpp et al., doi: 10.1016/j.cell.2009.12.001).

- Figure 3B: it is interesting that for some TFs, the growth rate is less than 1 when the TF number equals the physiological concentration as measured in Schmidt et al. Could the authors provide some explanations? My intuition is that the growth conditions could be different between this work and Schmidt et al., but I think this could be clearly stated in the text.

- Figure 4C: the curve for 1 TF is confusing for me. Could the authors explain why there is no titration effect? I could not find the information as to where the authors have put the PzntA and PDL5 promoters. But even the authors have put them into the genome, the promoter copy number should still be larger than one, and there will be less than 1 TF per binding site. The promoters cannot be fully repressed/activated by the regulators (Brewster et al., doi: 10.1016/j.cell.2014.02.022). However, there is still a 10 fold activation similar to the other TF numbers.

- Native and synthetic ZntR promoters: what are the basal expression levels for the two promoters? PDL5 has a better -10 box -- is it going to have a higher basal expression and is it going to affect the activation fold by Zinc?

- Figure 4D: what is the promoter used here? Is it a synthetic promoter? Please clarify.

- Figure 4E: the mathematical model needs more explanations. I am not sure how the model is related to the experimental results in Figure 4C. Do authors use PzntA and PDL5 to drive the expression of ZntA in their cell strains? If ZntA is controlled by the WT promoter, what will happen? The authors have stated that alpha_a > alpha in the main text, but I think this is not the case in figure 4F.

- Figure 5C: what is effective TF? I could not find this information in the caption or main text. I would suggest the authors explain the term or switch it back to the TF number.

- Kinetic model for ZntR regulation: the authors have used a mean-field theory model. Please justify why it will work for low TF numbers, especially when the TF number is comparable with the promoter number. In this scenario, the Pon and Poff of the promoters will depend on its total copy number and the "retroactivity" is non-negligible. In table S1, please include values of all kinetic parameters.

- Usage of mCherry: recently a report claims that the mCherry fluorescence reporter contains a short functional protein isoform (https://doi.org/10.1101/2021.12.07.471677). Could the authors comment on how possible technical caveats of mCherry fluorescence reporter would affect the interpretation of their results?

Minor comments:
- Page 3 line 11: "etc."
- Figure 2C: what is the shaded area?
- Figure 2 caption: "et al." should be used instead of "et. al."
- Figure 3 caption: I believe it should be "in the clustergram in Fig 3A"
- Figure 3B: please increase the font size of gene names
- Figure 3B: what are the black dots? Please clarify.
- Figure 5C: do authors include the regulatory curves for +12.5? If so, what is the color?
- Figure 5C caption: "and" is italic.
- Figure 5E: the symbol for BS4 in the figure legend is not right.
- Estimation of calibration factor: line 2, it should be "TF-mCherry".
- Estimation of calibration factor: please include temperature information in this part.
- Figure S1 E&F: Please include error bars for the data points.
- Figure S1G: what is the unit for growth rate in the y axis?
- Figure S2: what is alpha2?
- Figure S3: what are the error bars and how many replicates are done?
- Measurements of calibration factor: format problem after equation (3).
- Measurements of calibration factor: the third line after equation (3), "up to".
- Figure S4B: what are the colors?
- Figure S4 caption: "For most TFs".
- Figure S5: there are multiple red/purple lines in the figure and I am not sure what they are.
- Figure S6A: it should be "library strain H-NS" in the figure title.
- Figure S6B: "mCherry" in the figure title and y-axis label.
- Figure S7: please include aTC concentrations in the plots.

Reviewer #1:

Predicting the regulatory function of a transcription factor (TF) is challenging. Although activity is influenced by concentration, several other factors, including co-factors like small molecules or metals, degree of phosphorylation, associated proteins, and the interconnected nature of regulatory networks, where network motifs like feedforward loops are common, can also play a role. To investigate the influence of a TF's concentration in a manner that limits the influence of these confounding factors, Parisutham et al. have generated a library of close to 200 *E. coli* strains producing titrable TFs, where TF concentration can be manipulated by addition of a chosen amount of inducer (tetracycline). This set-up allows to study the regulatory function of a TF in a way that makes it more independent of the network properties. Furthermore, TF levels can be measured by recording the fluorescence emitted by mCherry which is fused to the TF of interest. They present two practical examples in support of their approach. The first example deals with a promoter whose activity is dependent on a TF that binds a heavy metal. In this class of promoters, the spacing between the -10 and -35 binding sequences for the sigma factor of the RNA polymerase (RNAP) is not optimal. A metal associates with a TF that is already bound between the -10 and -35 sequences, thus re-orienting the DNA in a way that will now allow the binding of the RNAP holoenzyme. In the experimental set-up, a reporter is used to measure gene activity and the concentrations of both the TF and the metal co-factor can be controlled directly by the experimenter. This strategy was successful in unveiling a formerly unknown aspect of the regulatory process, where increasing amounts of metal lead to a shift in the TF function, from repressor to activator. This subtle behavior would have been difficult to observe with conventional approaches. Unexpectedly, this approach also revealed that this transition was mostly independent from TF concentration. In the second example, the authors explore the case of paralogous TFs, i.e., those that arose from gene duplication and often recognize similar binding sequences. These TFs frequently have overlapping regulons and it is unclear if they do in fact act differently on their shared targets or if they have independent target genes. Importantly, the experimental strategy allows the independent titration of two paralogous TFs and these questions can be investigated in a controlled manner. Specifically, by studying the paralogs GalR and GalS, which bind to similar sequences, it was possible to uncover differential regulation, where one TF acts more frequently as an activator and the other as a repressor. In general, the data support the authors 'claim that their library is going to be a useful resource for studies of mechanisms of transcriptional regulation and the examples are well chosen. I only have a few comments.
Specific points:
1)Although the addition of mCherry is useful to quantify the amount of TF produced in the induction system, it is important to emphasize that the addition of mCherry may change the

properties of the TF, e.g., by making it more stable or interfering with interaction with certain binding partners. It may for instance explain why the ZntR-mCherry fusion acts as a repressor in the absence of Zn. I don't think that this will limit the use of the library in most cases, but it would be helpful to comment on this possibility somewhere in the text

Response: We agree with the reviewer that tagging TFs with mCherry could have non-generalizable impact on different TFs. However, because we have all the TFs integrated at the common genetic locus with a linker separating the TF and the mCherry, one can easily develop systematic ways to change the fluorophore or introduce a stop codon to express an untagged TF. In fact, in this revision we have remade the entire library thanks to a comment in these reports about an internal start codon in our mCherry. We have removed that codon from the entire library and retaken the data for this submission. We also now include, in the appendix section, a guide to the readers to use the library and systematically introduce any desired changes to the fluorescent tag (See Appendix.1. “Systematic swapping of the fluorophore”).

2)In terms of generalizing the use of the library to as many TFs as possible, it is likely that certain classes of TFs are going to pose specific problems that will need to be addressed. For instance, the activity of response regulators in two component systems is controlled by phosphorylation. Titrating the response regulator without similarly modulating the concentration of the cognate histidine kinase is unlikely to produce meaningful data. The same is true for certain TF that rely on cofactors. In the example chosen, the co-factor is a metal (Zn), whose concentration can be modified by adding specific amount to the medium. For other factors, like for cAMP or certain metabolites, it might not be as easy.

Response: We agree with the reviewer’s concern, previously, our lab has successfully demonstrated the potentials of a phosphorylated-TF (*cpxR*) where no such modifications was indeed necessary (Guharajan et al (2021)). CpxR might be a special case as it activates the expression of its own kinase but may not be generalized for other kinase and need careful characterization. We highlight in the supplementary Fig S6 on the limitations in our ability to study phosphorylated TFs. We have also listed the percentage of TFs in our library that require modifications such as intracellular co-factors or phosphorylation (Fig S1 (A-C)). As we have shown with our own examples, any genetic changes can be easily combined with our library for instance, we can P1 any single gene knockouts from kieo library to our TF library (as demonstrated with our GalR/GalS system) or over-express cognate histidine kinases to achieve phosphorylation of the target TFs. We are also looking forward to making modifications to the phosphorylated TFs in the future. We also want to highlight that according to Schmidt et al proteomic analysis not many of the TFs are prone to modifications.

Minor:
p.6, first paragraph of discussion, letter missing, it should be "Pseudomonas aeruginosa"

Response: We have fixed this.

p.6, last paragraph, should be "complementary tool" instead of "complimentary" unless the authors want to emphasize the fact that the use of their library is free of charge.
Response: We have fixed this error. We will be happy to distribute it free of charge to different labs interested in using our library. We have added a note about the free distribution in the manuscript.

Reviewer #2:

Review, Parisutham et al.

Summary

In this work, the authors create a library of *E. coli* strains where each *E. coli* transcription factor has been fused to mCherry and put under the control of the Ptet promoter. In conjunction with appropriate fluorescence calibration, these strains can be used to characterize the gene regulatory function for most *E. coli* transcription factors under different growth conditions and for multiple gene targets.

General remarks

Characterizing the gene regulation functions for transcription factors is fundamental to obtaining a quantitative understanding of gene regulation. Even this has been achieved, at different levels of detail, for a select set of endogenous transcription factors, we lack a quantitative understanding of most transcription factors for any biological system. Tools like the one presented in this work are a valuable addition to the arsenal of methods available to scientists to study new transcription factors and better understand gene regulation.

Major comments

- It has been reported that fusions of mCherry and other fluorescent proteins can form spurious fluorescent foci (Landgraf et al., Nature methods, 2012). The authors encountered foci in some of their low-induction strains. Is it possible to see foci in the high-induction strains in the low concentration regime?

Response: Surprisingly, we only see 3 total strains that show strong foci which show those foci at all induction conditions. We never observe foci for other TFs at any inducer concentration that we have seen.

- What is the rationale behind using mCherry, instead of other fluorescent proteins that may show lower foci formation?

Response: Overall there is room for about 3 non-overlapping fluorophores in the usable spectrum (roughly speaking: blue, yellow, red). Blue is a notoriously difficult color for *E.* coli; the excitation light is extremely toxic, the autofluorescence is highest and practically speaking we have had the hardest time quantifying signals from those fluors (*i.e.* getting smooth titration curves from standards). Based on this, we have chosen yellow for reporter and red for TFs. We have used this in several studies without observing issues. The choice of mCherry also makes this library compatible with the previously developed Zaslaver's transcriptional reporter library (Zaslaver 2004} and Binding location sweep library'' from our lab that uses GFP and YFP, respectively. However, because we have all the TFs integrated at the common genetic locus, we can easily develop a systematic way to change the fluorophore or remove all the fluorophore and compare TF functionality based on the choice of the tag. We have extended the appendix to include the possible ways to re-engineer the TF library in a systematic way to have a different fluorescent protein. We have also demonstrated during the review process how we replaced the $mCherry^{wt}$ with the $mCherry^{M10L}$ variant to account for the additional isoform that is otherwise present in $mCherry^{wt}$

- What is the detection threshold for their method, particularly the plate reader method?

Response: In the revised manuscript we re-acquired all fluorescence measurements in the microscope instead of the plate reader. The detection threshold for our microscopy settings is approximately 5 TFs, we know from negative controls that even control cells without TF-mCherry fusions the fluctuations in total intensity can be as large as 5 TFs. In general, even when our library strains are uninduced, the leaky levels surpass 5 TFs per cell.

Minor comments:

- "Fourth cluster in purple" should be "The fourth cluster in purple"

Response: We fixed it.

- "aerugino" should be "aeruginosa"

Response: We fixed this.

- "Predicting the (...) is not possible". Maybe not now, but maybe in the future.

Response: We fixed this.

- The images are helpful and well organized, but there are multiple cases where it is tough to read the labels. Consider adjusting the font size, so it is legible when in paper format. Some examples:
• Figure 2B, inset, axis labels

Response: We fixed this.

• Figure 3B, Top, legend; Bottom, gene names

Response: We fixed this.
• Figure S1D-H, multiple cases

Response: We fixed this.

- Could not find references in the main text for Figure S6 or S7.
Response: Thanks for pointing these out. Figure S6 and S7 are Fig S7 and S8 in the revised manuscript. We now reference Fig S7 in Page 2 under the section: "Physiological effects of TF titration" and Fig S8 in the legend for Fig 3

Reviewer #3:

In "Tunable Transcription Factor Library for Robust Quantification of Regulatory Properties in *E. coli*", the authors present a library of *E. coli* cell strains that enable precise control of TF concentrations. In each library strain, the authors knockout one TF gene and introduce a titratable TF copy into the genome. The TF abundances are determined using the mCherry fluorescence reporter. The authors show that this newly generated library can be combined with rationally designed promoters to uncover the regulatory functions of individual TFs in two case studies.

This work could have a large impact on the field. Although studies on individual model TFs have been fruitful in the past decade, response functions of most TFs in *E. coli* are still unexplored and the quantitative understanding of their regulatory behavior is largely hindered by the lacking of single-cell level tools. The presented library covers 194 TFs in *E. coli*. It could potentially serve as a standard for quantitative studies on TF functions and facilitate the genetic part design for complex functions. The authors are encouraged to include a statement about how the cell strains will be available to the community.

The manuscript is well-structured and contains a large amount of quantitative data. By fine-tuning the TF number and promoter structure, the authors provide some novel insights on the regulatory functions of Zinc-responsive TF ZntR and isorepressors GalR and GalS. However, I would like to invite the authors to address several technical issues that have undermined the strength of the current manuscript. I am also not very convinced about the authors' modeling approach. Detailed comments are given below.

Major comments
- Figure 2C: besides ArgR, there are still large differences in calibration factors for other TFs. A bit of text to explain the possible reasons would be helpful. The authors have claimed that the calculation of v depends heavily on experimental settings (page 3 line 11). Do they use the same experimental settings for all 10 TFs? If so, please extend the "Estimation of calibration factor" part to include more information such as the exposure time.

Response: We remade the library by replacing the wild type mCherry with mCherry(M10L) and the calibration factors measured in the modified mCherry variant have tightened significantly especially for those expressing high mCherry levels such as ArgR, there is now less difference in the calibration factors. We suspect this is due to the elimination of short isoform translated products mixed with full length TF-mCherry fusions. We have updated the manuscript with the calibration factors measured from the newer mCherry variant. However, we do expect that in some cases the fluorescence properties of the fluor may be impacted by the fused TF particularly when the TF exists in multimeric forms or need different co-factors/modifications to function.

- Figure 2E: how the authors estimate the absolute TF number per cell is not clear enough for me. I understand that the authors could measure TF conc. using plate reader but how do they convert the TF conc. to TF absolute number per cell? Do the authors assume the cell sizes for the library strains at maximal induction are all the same? From figure 3B, the growth rates for the strain library at maximal induction are very different and there should be changes in the cell size (Klumpp et al., doi: 10.1016/j.cell.2009.12.001).

Response: This is an excellent point. We had originally assumed that growth differences would be relatively minor and only impact a small percent of strains. Rethinking this, we were unsatisfied with these assumptions. Hence, we remade all mCherry measurements in the microscope itself where we can explicitly measure fluorescence per cell by segmenting fluorescent microscope images and identifying individual cells. To ease the number of samples we reduced the number of aTC concentration from 7 to 4 and re-measured all the TFs mCherry measurement in the microscope and normalized it with the mean of the calibration factor measured for the 9 strains.

- Figure 3B: it is interesting that for some TFs, the growth rate is less than 1 when the TF number equals the physiological concentration as measured in Schmidt et al. Could the authors provide some explanations? My intuition is that the growth conditions could be different between this work and Schmidt et al., but I think this could be clearly stated in the text.

Response: Yes, the growth condition between our work and Schmidt et al work is different. Our M9-glucose media have no trace element, and we grow cells in a 37 °C incubator shaking at 250 rpm in 96 well plate whereas in the work of Schmidt et al, the M9-media is supplemented with trace element and thiamine, and they grow cells in a 500mL flask shaking at 300 rpm. Apart from the changes in growth conditions, the mCherry tag could also reduce or alter the activity of the TF. We have explicitly discussed the growth condition differences between our work and the work of Schmidt et al under the section "Estimation of absolute TF numbers for the entire library". The major goal in comparing to the physiological concentrations is to highlight that we could titrate TF copy number better than just the conventional way of controlling protein concentration by changing the growth rates.

- Figure 4C: the curve for 1 TF is confusing for me. Could the authors explain why there is no titration effect? I could not find the information as to where the authors have put the PzntA and PDL5 promoters. But even the authors have put them into the genome, the promoter copy number should still be larger than one, and there will be less than 1 TF per binding

site. The promoters cannot be fully repressed/activated by the regulators (Brewster et al., doi: 10.1016/j.cell.2014.02.022). However, there is still a 10 fold activation similar to the other TF numbers.

Response: This is an insightful point, particularly in conjunction with the question regarding detection limit. The labels on these bins actually (in error) referred to the left edge of the bin, rather than the center. Still it was a bit overambitious for us to label low signal cells with this precision. In the revised manuscript we have corrected this and the lowest bins center is now at 12 TFs where we feel more confident that we can distinguish which bin a cell should belong to. We have also included supplementary Fig S3, to show the raw data of TF copy number versus fold-change for a given zinc concentration. It will be more evident from the plot that the expression is solely dependent on the zinc concentration and not on the TF copy number.

The promoters PzntA and PDL5 are on SC101 low copy number plasmids, this is mentioned in the materials and methods section and is now explicitly stated in the main text section in Page 6 (highlighted in red at the end of the paragraph) of the paper. In our lab we have measured the copy number of these plasmids to be roughly 3-4 per cell (consistent with other measurements such as Binshal *et. al*, Nat. Com. 2021). We suspect that in that range the titration effect will be very difficult to measure due to the similarity between the copy number and the lower edge of our detection limit.

(A) Fold change as a function of TF copy number

(i) P_{zntA} promoter

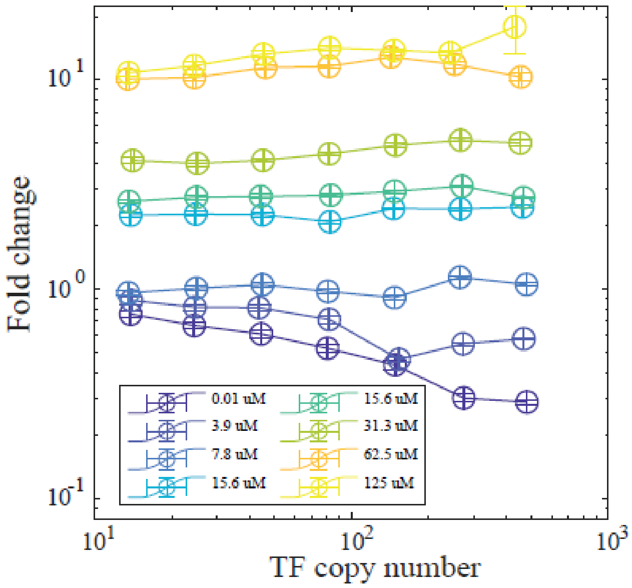

It is surprising that the promoter response is very robust and almost independent of the TF copy number in the presence of zinc. It is possible that this is an indication of strong stabilization of RNA polymerase at the promoter by ZntR which serves to effectively lower the K_D of the TF (see the effect of beta on chi in Fig 1 of Guharajan *et al*, Cell reports 2021, for instance). However, we have not explored the regulatory properties of ZntR deep enough to comment on this for sure. When the same binding site is downstream of the promoter or on a stronger promoter element we could see a TF dependent regulation with or without zinc (Fig 4D) similar to that one described in Brewster et al., doi: 10.1016/j.cell.2014.02.022.

- Native and synthetic ZntR promoters: what are the basal expression levels for the two promoters? PDL5 has a better -10 box -- is it going to have a higher basal expression and is it going to affect the activation fold by Zinc?

Response: This is correct, the basal expression for P_{DL5} is higher than P_{zntA} . This is now explicitly shown in Fig 4D. Indeed, this also has an impact on the regulation by ZntR. We have updated the discussion of the model and the presentation of the results considerably and hopefully the ideas are significantly more clear now.

- Figure 4D: what is the promoter used here? Is it a synthetic promoter? Please clarify.

Response: Fig 4D (which is now Fig 4E) uses the PDL5 promoter with a ZntR binding site inserted at +16.5. We have updated the figure to explicitly show that the promoter in this case is PDL5.

- Figure 4E: the mathematical model needs more explanations. I am not sure how the model is related to the experimental results in Figure 4C. Do authors use PzntA and PDL5 to drive the expression of ZntA in their cell strains? If ZntA is controlled by the WT promoter, what will happen? The authors have stated that alpha_a > alpha in the main text, but I think this is not the case in figure 4F.

Response: ZntA is always expressed by the wild type P_{ZntA} promoter from the chromosome. The ZntA expression is not altered in the experiments. The description and integration of the modeling section has been entirely redone. We hope that the results and insight generated from the model are now more clear. For instance, we have gotten rid of the alpha_a

notation and now use a more generic nomenclature with “r” and “r_0” without an implicit assumption about the relative strength of the two effects.

- Figure 5C: what is effective TF? I could not find this information in the caption or main text. I would suggest the authors explain the term or switch it back to the TF number.

Response: Thanks for pointing to it. Effective TF number is the TF copy number normalized by the binding site affinity (more precisely: exp(-Delta E)/Nns in thermodynamic lingo). We have added this definition to Page 9 just below equation 1. Effective TF number is an easier way to make direct comparison between different TFs as it accounts for both the copy number of the TF and the affinity of the site used and enables us to visualize the theory with only one free parameter, the FCmax.

- Kinetic model for ZntR regulation: the authors have used a mean-field theory model. Please justify why it will work for low TF numbers, especially when the TF number is comparable with the promoter number. In this scenario, the Pon and Poff of the promoters will depend on its total copy number and the "retroactivity" is non-negligible. In table S1, please include values of all kinetic parameters.

Response: We have re-written the kinetic model giving more details about all the assumptions and the kinetic parameters used. We have also revised the estimation of TF number for those bins (previously the label came from the left edge of the bin rather than the center). Given this, we expect there still could be a minimal impact of promoter copy number on the data. The net result of such an impact would be a systematic under-measurement of the fold-change (both above and below 1) for the lowest TF bin. This may indeed be influencing our results; however, we do not seek exact quantitative matching with the model, only to gain qualitative insights into the observed behaviors.

We only have two free parameters in the model (r_0 and r) and we inferred values for k_{on}/k_{off} (from the binding energy measured in Fig 4E) and ɣ (from the growth rate). For r_0 , we estimate the proportions based on the constitutive values measured in the different reporters we used. Since we do not know several details on the intracellular zinc concentration or the affinity constants or rate constant for zinc transport, we convolved all these terms as “effective zinc concentration” and plot fold-change as a function of effective TF concentration. We use TF copy numbers between 10 and 300 for the kinetic model. We hope the new amendments and details will be convincing.

- Usage of mCherry: recently a report claims that the mCherry fluorescence reporter contains a short functional protein isoform (https://doi.org/10.1101/2021.12.07.471677). Could the authors comment on how possible technical caveats of mCherry fluorescence reporter would affect the interpretation of their results?

Response: We tested for the presence of internal isoform for the version of mCherry in our library strain by introducing stop codon in the linker sequence and measured for the read-through transcription. Like the above reference we saw significant read-through from the short isoform in our constructs. The extra transcript affects our ability to accurately quantify the TF copy number but will not affect the qualitative features of our data. Hence, we reconstructed the downstream fusion by removing the ATG at position 1 and substituting methionine at 10th position with a Leucine. The manuscript now has all data re-taken with the newer library except for the data describing the GalR and GalS. We have instead re-normalized the TF copy number by estimating the fraction of the additional transcript in the older mCherry version. Please refer to SI Fig 6 for the corrections and normalization. In general, the strains expressing higher range of mCherry signals are largely affected by the internal variant than the strains expressing lower range of mCherry signal.

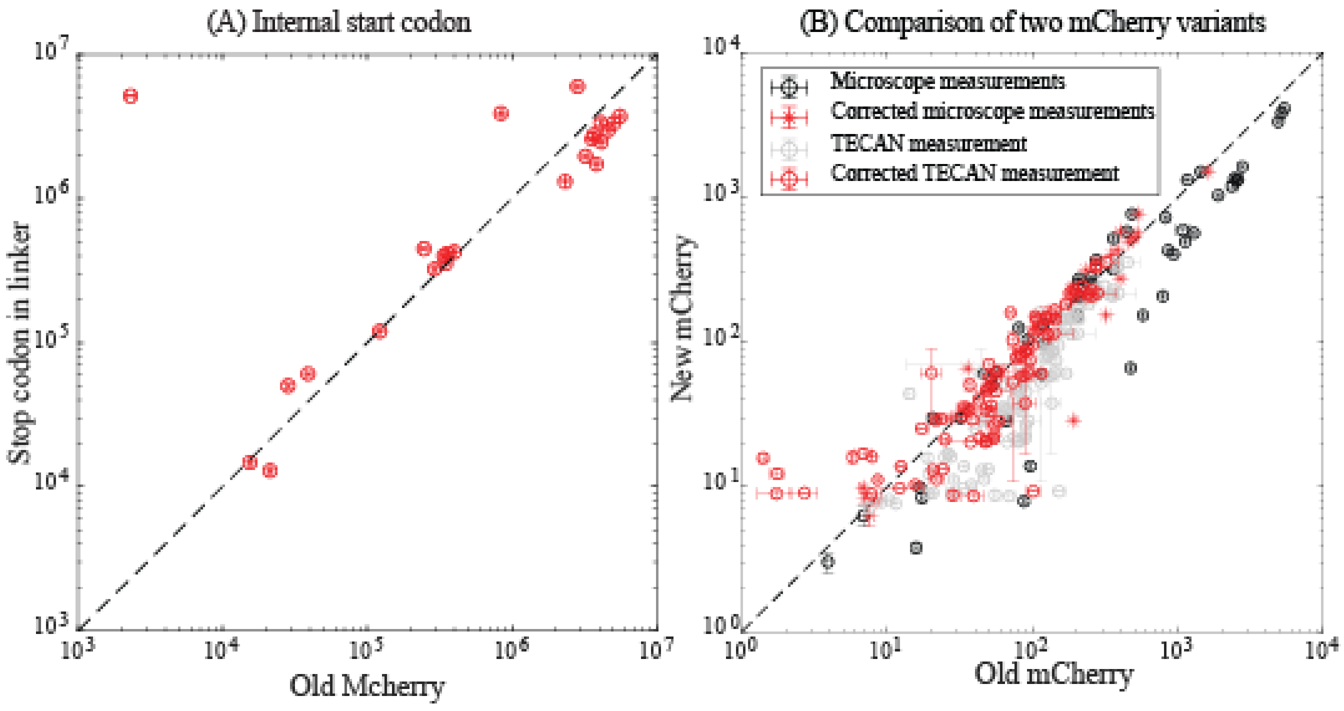

Minor comments:
- Page 3 line 11: "etc."

Response: This has been fixed.

- Figure 2C: what is the shaded area?

Response: Shaded area is the mean and standard deviation of the calibration factor. We have updated the legend.
- Figure 2 caption: "et al." should be used instead of "et. al."

Response: This has been fixed.

- Figure 3 caption: I believe it should be "in the clustergram in Fig 3A"

Response: This has been fixed.

- Figure 3B: please increase the font size of gene names

Response: Done

- Figure 3B: what are the black dots? Please clarify.

Response: Black dots are the growth rate of the knockouts. For clarity we replaced the black dots with red dashed lines and update the legend with the information.

- Figure 5C: do authors include the regulatory curves for +12.5? If so, what is the color?

Response: Yes, the legends are updated to show that

- Figure 5C caption: "and" is italic.

Response: This has been fixed.

- Figure 5E: the symbol for BS4 in the figure legend is not right.

Response: This has been fixed.

- Estimation of calibration factor: line 2, it should be "TF-mCherry".
Response: This has been fixed.

- Estimation of calibration factor: please include temperature information in this part.
Response: This has been fixed.

- Figure S1 E&F: Please include error bars for the data points.

Response: We do not have these figures in the revised manuscript as we remade all the measurements in the microscope and no longer need the microplate measurements.

- Figure S1G: what is the unit for growth rate in the y axis?

Response: The unit is (hr-1). We have updated the unit. We have rearranged the supplementary figures a little bit. This figure is now part of Fig S2.

- Figure S2: what is alpha2?

Response: This is now S3 in the revised manuscript We initially used alpha2 in place of alpha. We fixed the inconsistency. We now call this term as “r0” to account for the basal transcription rate.

- Figure S3: what are the error bars and how many replicates are done?

Response: 3 replicates are done per each experiment. Single cell fold-change is calculated independently for the three experiments and all the single cells data are binned. The errorbars are the standard error in each bin.

- Measurements of calibration factor: format problem after equation (3).

Response: Fixed

- Measurements of calibration factor: the third line after equation (3), "up to".

Response: Fixed

- Figure S4B: what are the colors? - Figure S4 caption: "For most TFs".

Response: We do not have this figure in the updated manuscript as we no longer use this variant of mCherry.

- Figure S5: there are multiple red/purple lines in the figure and I am not sure what they are.

Response: Those are different promoters regulated by the same TF. We have updated the legend and caption with more information on the type of promoter and the corresponding

TF. In the revised manuscript this figure is S6.

- Figure S6A: it should be "library strain H-NS" in the figure title.

- Figure S6B: "mCherry" in the figure title and y-axis label.

Response: We fixed the above two comments. In the revised manuscript this figure is S7

- Figure S7: please include aTC concentrations in the plots.

Response: We have added aTC concentration in all the plots.

Thank you for sending us your revised manuscript. We have now heard back from reviewer #3 who was asked to evaluate your revised study. As you will see below, their concerns have been satisfactorily addressed and they are supportive of publication. I am glad to inform you that we can soon accept the study for publication, pending some minor editorial issues listed below.

REFEREE REPORTS

Reviewer #3:

The authors have adequately addressed the questions I raised in previous review. I have no further comments and recommend acceptance of the manuscript for publication.

The authors have made all requested editorial changes.

ACCEPTED **13th May 2022**

Thank you again for sending us your revised manuscript. We are now satisfied with the modifications made and I am pleased to inform you that your paper has been accepted for publication.

EMBO PRESS

YOU MUST COMPLETE ALL CELLS WITH A PINK BACKGROUND ➔

PLEASE NOTE THAT THIS CHECKLIST WILL BE PUBLISHED ALONGSIDE YOUR PAPER

Corresponding Author Name: Robert C Brewster
Journal Submitted to: Molecular Systems Biology
Manuscript Number: MSB-2021-10843

Reporting Checklist For Life Sciences Articles (Rev. June 2017)

This checklist is used to ensure good reporting standards and to improve the reproducibility of published results. These guidelines are consistent with the Principles and Guidelines for Reporting Preclinical Research issued by the NIH in 2014. Please follow the journal's authorship guidelines in preparing your manuscript.

A- Figures
1. Data
The data shown in figures should satisfy the following conditions:

- the data were obtained and processed according to the field's best practice and are presented to reflect the results of the experiments in an accurate and unbiased manner.
- figure panels include only data points, measurements or observations that can be compared to each other in a scientifically meaningful way.
- graphs include clearly labeled error bars for independent experiments and sample sizes. Unless justified, error bars should not be shown for technical replicates.
- if n< 5, the individual data points from each experiment should be plotted and any statistical test employed should be justified
- Source Data should be included to report the data underlying graphs. Please follow the guidelines set out in the author ship guidelines on Data Presentation.

2. Captions

Each figure caption should contain the following information, for each panel where they are relevant:

- a specification of the experimental system investigated (eg cell line, species name).
- the assay(s) and method(s) used to carry out the reported observations and measurements
- an explicit mention of the biological and chemical entity(ies) that are being measured.
- an explicit mention of the biological and chemical entity(ies) that are altered/varied/perturbed in a controlled manner.

- the exact sample size (n) for each experimental group/condition, given as a number, not a range;
- a description of the sample collection allowing the reader to understand whether the samples represent technical or biological replicates (including how many animals, litters, cultures, etc.).
- a statement of how many times the experiment shown was independently replicated in the laboratory.
- definitions of statistical methods and measures:
  - common tests, such as t-test (please specify whether paired vs. unpaired), simple χ2 tests, Wilcoxon and Mann-Whitney tests, can be unambiguously identified by name only, but more complex techniques should be described in the methods section;
  - are tests one-sided or two-sided?
  - are there adjustments for multiple comparisons?
  - exact statistical test results, e.g., P values = x but not P values < x;
  - definition of 'center values' as median or average;
  - definition of error bars as s.d. or s.e.m.

Any descriptions too long for the figure legend should be included in the methods section and/or with the source data.

In the pink boxes below, please ensure that the answers to the following questions are reported in the manuscript itself. Every question should be answered. If the question is not relevant to your research, please write NA (non applicable). We encourage you to include a specific subsection in the methods section for statistics, reagents, animal models and human subjects.

USEFUL LINKS FOR COMPLETING THIS FORM

http://www.antibodypedia.com
http://1degreebio.org
http://www.equator-network.org/reporting-guidelines/improving-bioscience-research-repor
http://grants.nih.gov/grants/olaw/olaw.htm
http://www.mrc.ac.uk/Ourresearch/Ethicsresearchguidance/Useofanimals/index.htm
http://ClinicalTrials.gov
http://www.consort-statement.org
http://www.consort-statement.org/checklists/view/32-consort/66-title
http://www.equator-network.org/reporting-guidelines/reporting-recommendations-for-tumo
http://datadryad.org
http://figshare.com
http://www.ncbi.nlm.nih.gov/gap
http://www.ebi.ac.uk/ega
http://biomodels.net/
http://biomodels.net/miriam/
http://jjj.biochem.sun.ac.za
https://osp.od.nih.gov/biosafety-biosecurity-and-emerging-biotechnology/
http://www.selectagents.gov/

B- Statistics and general methods

Please fill out these boxes ➔ (Do not worry if you cannot see all your text once you press return)

1.a. How was the sample size chosen to ensure adequate power to detect a pre-specified effect size?	All data has is taken in at least triplicate, single cell data for fluorescence typically contains over a thousand cells.
1.b. For animal studies, include a statement about sample size estimate even if no statistical methods were used.	NA
2. Describe inclusion/exclusion criteria if samples or animals were excluded from the analysis. Were the criteria pre-established?	NA
3. Were any steps taken to minimize the effects of subjective bias when allocating animals/samples to treatment (e.g. randomization procedure)? If yes, please describe.	NA
For animal studies, include a statement about randomization even if no randomization was used.	NA
4.a. Were any steps taken to minimize the effects of subjective bias during group allocation or/and when assessing results (e.g. blinding of the investigator)? If yes please describe.	NA
4.b. For animal studies, include a statement about blinding even if no blinding was done	NA
5. For every figure, are statistical tests justified as appropriate?	Yes
Do the data meet the assumptions of the tests (e.g., normal distribution)? Describe any methods used to assess it.	Yes
Is there an estimate of variation within each group of data?	Standard error of the mean for multiple data with multiple trials.

Is the variance similar between the groups that are being statistically compared?	Yes

C- Reagents

6. To show that antibodies were profiled for use in the system under study (assay and species), provide a citation, catalog number and/or clone number, supplementary information or reference to an antibody validation profile. e.g., Antibodypedia (see link list at top right), 1DegreeBio (see link list at top right).	NA
7. Identify the source of cell lines and report if they were recently authenticated (e.g., by STR profiling) and tested for mycoplasma contamination.	NA

* for all hyperlinks, please see the table at the top right of the document

D- Animal Models

8. Report species, strain, gender, age of animals and genetic modification status where applicable. Please detail housing and husbandry conditions and the source of animals.	NA
9. For experiments involving live vertebrates, include a statement of compliance with ethical regulations and identify the committee(s) approving the experiments.	NA
10. We recommend consulting the ARRIVE guidelines (see link list at top right) (PLoS Biol. 8(6), e1000412, 2010) to ensure that other relevant aspects of animal studies are adequately reported. See author guidelines, under 'Reporting Guidelines'. See also: NIH (see link list at top right) and MRC (see link list at top right) recommendations. Please confirm compliance.	NA

E- Human Subjects

11. Identify the committee(s) approving the study protocol.	NA
12. Include a statement confirming that informed consent was obtained from all subjects and that the experiments conformed to the principles set out in the WMA Declaration of Helsinki and the Department of Health and Human Services Belmont Report.	NA
13. For publication of patient photos, include a statement confirming that consent to publish was obtained.	NA
14. Report any restrictions on the availability (and/or on the use) of human data or samples.	NA
15. Report the clinical trial registration number (at ClinicalTrials.gov or equivalent), where applicable.	NA
16. For phase II and III randomized controlled trials, please refer to the CONSORT flow diagram (see link list at top right) and submit the CONSORT checklist (see link list at top right) with your submission. See author guidelines, under 'Reporting Guidelines'. Please confirm you have submitted this list.	NA
17. For tumor marker prognostic studies, we recommend that you follow the REMARK reporting guidelines (see link list at top right). See author guidelines, under 'Reporting Guidelines'. Please confirm you have followed these guidelines.	NA

F- Data Accessibility

18: Provide a "Data Availability" section at the end of the Materials & Methods, listing the accession codes for data generated in this study and deposited in a public database (e.g. RNA-Seq data: Gene Expression Omnibus GSE39462, Proteomics data: PRIDE PXD000208 etc.) Please refer to our author guidelines for 'Data Deposition'.<br><br>Data deposition in a public repository is mandatory for:<br>a. Protein, DNA and RNA sequences<br>b. Macromolecular structures<br>c. Crystallographic data for small molecules<br>d. Functional genomics data<br>e. Proteomics and molecular interactions	Done
19. Deposition is strongly recommended for any datasets that are central and integral to the study; please consider the journal's data policy. If no structured public repository exists for a given data type, we encourage the provision of datasets in the manuscript as a Supplementary Document (see author guidelines under 'Expanded View' or in unstructured repositories such as Dryad (see link list at top right) or Figshare (see link list at top right).	NA
20. Access to human clinical and genomic datasets should be provided with as few restrictions as possible while respecting ethical obligations to the patients and relevant medical and legal issues. If practically possible and compatible with the individual consent agreement used in the study, such data should be deposited in one of the major public access-controlled repositories such as dbGAP (see link list at top right) or EGA (see link list at top right).	NA
21. Computational models that are central and integral to a study should be shared without restrictions and provided in a machine-readable form. The relevant accession numbers or links should be provided. When possible, standardized format (SBML, CellML) should be used instead of scripts (e.g. MATLAB). Authors are strongly encouraged to follow the MIRIAM guidelines (see link list at top right) and deposit their model in a public database such as Biomodels (see link list at top right) or JWS Online (see link list at top right). If computer source code is provided with the paper, it should be deposited in a public repository or included in supplementary information.	Done

G- Dual use research of concern

22. Could your study fall under dual use research restrictions? Please check biosecurity documents (see link list at top right) and list of select agents and toxins (APHIS/CDC) (see link list at top right). According to our biosecurity guidelines, provide a statement only if it could.	NA
